# Supplementary material for: Spatial epigenome–transcriptome co-profiling of mammalian tissues
Source: Nature. 2023 Mar 15;616(7955):113–22. doi: 10.1038/s41586-023-05795-1 (PMC10076218; doi:10.1038/s41586-023-05795-1)
Supplement: Supplementary file 1 — Supplementary Figs. 1–14, Tables 2–7 and statistics and reproducibility. [file 41586_2023_5795_MOESM1_ESM.pdf]

nature portfolio

https://doi.org/10.1038/s41586-023-05795-1

Supplementary information

Spatial epigenome–transcriptome co-profiling of mammalian tissues

In the format provided by the authors and unedited

Spatial epigenome-transcriptome co-profiling of mammalian tissues

Di Zhang[1,21], Yanxiang Deng[1,2,20,21,*], Petra Kukanja[3], Eneritz Agirre[3], Marek Bartosovic[3], Mingze Dong[4,5], Cong Ma[6], Sai Ma[7], Graham Su[1,2], Shuozhen Bao[1], Yang Liu[1,2], Yang Xiao[8], Gorazd B. Rosoklija[9,10,11], Andrew J. Dwork[9,10,11,12], J. John Mann[9,10,13], Kam W. Leong[8,14], Maura Boldrini[9,10], Liya Wang[15], Maximilian Haeussler[16], Benjamin J. Raphael[6], Yuval Kluger[4,5,17], Gonçalo Castelo-Branco[3,18,*], Rong Fan[1,2,4,19,*]

[1]Department of Biomedical Engineering, Yale University, New Haven, CT 06520, USA
[2]Yale Stem Cell Center and Yale Cancer Center, Yale School of Medicine, New Haven, CT 06520, USA
[3]Laboratory of Molecular Neurobiology, Department of Medical Biochemistry and Biophysics, Karolinska Institutet, Stockholm, Sweden
[4]Department of Pathology, Yale University School of Medicine, New Haven, CT, USA
[5]Interdepartmental Program in Computational Biology and Bioinformatics, Yale University, New Haven, CT, USA
[6]Department of Computer Science, Princeton University, 35 Olden St, Princeton, NJ 08540, USA
[7]Klarman Cell Observatory, Broad Institute of MIT and Harvard, Cambridge, MA 02142, USA
[8]Department of Biomedical Engineering, Columbia University, USA
[9]Department of Psychiatry, Columbia University, New York, NY, 10032 USA
[10]Division of Molecular Imaging and Neuropathology, New York State Psychiatric Institute, New York, NY, 10032 USA
[11]Macedonian Academy of Sciences & Arts, Skopje, Republic of Macedonia
[12]Department of Pathology and Cell Biology, Columbia University, New York, NY, 10032 USA
[13]Department of Radiology, Columbia University, New York, NY, 10032 USA
[14]Department of Systems Biology, Columbia University Irving Medical Center, New York, NY 10032, USA
[15]AtlasXomics Inc., 290 Congress Ave, New Haven, CT 06519, USA
[16]Genomics Institute, University of California Santa Cruz, Santa Cruz, CA, USA
[17]Applied Math Program, Yale University, New Haven, CT, USA
[18]Ming Wai Lau Centre for Reparative Medicine, Stockholm node, Karolinska Institutet, Stockholm, Sweden
[19]Human and Translational Immunology Program, Yale School of Medicine, New Haven, CT 06520, USA
[20]Present address: Department of Pathology and Laboratory Medicine, Epigenetics Institute, Perelman School of Medicine, University of Pennsylvania, Philadelphia, PA 19104, USA
[21]These authors contributed equally.

*Corresponding authors. Email: rong.fan@yale.edu (R.F.), yanxiang.deng@pennmedicine.upenn.edu (Y.D.), goncalo.castelo-branco@ki.se (G.C.-B.)

Table of Contents

3 Supplementary Figs. 1-14
17 Statistics and Reproducibility
18 Supplementary Table 1 (legend included, table in separate Excel file)
19 Supplementary Tables 2-7

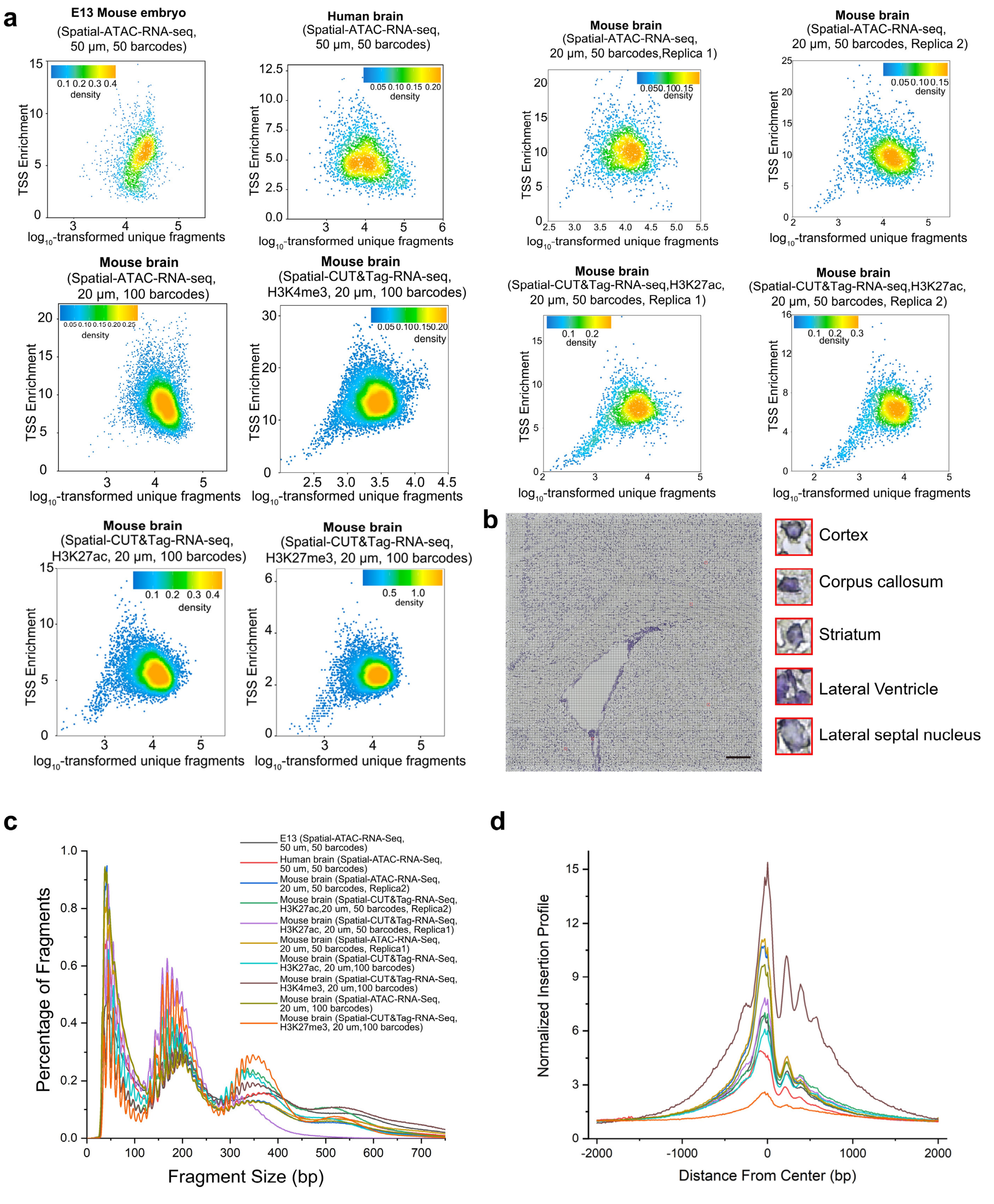

Supplementary Fig. 1 Quality control metrics for spatial-ATAC-RNA-seq and spatial-CUT&Tag-RNA-seq datasets. a, Scatterplots showing the TSS enrichment score vs unique nuclear fragments per pixel. **b,** Nissl-stained image from an adjacent tissue section of P21 mouse brain. The grid has an interval of 20 μm. Scale bar, 200 μm. **c,** The insert size distribution of ATAC or CUT&Tag fragments in spatial-ATAC-RNA-seq or spatial-CUT&Tag-RNA-seq. **d,** The enrichment of ATAC or CUT&Tag reads around TSSs in spatial-ATAC-RNA-seq or spatial-CUT&Tag-RNA-seq.

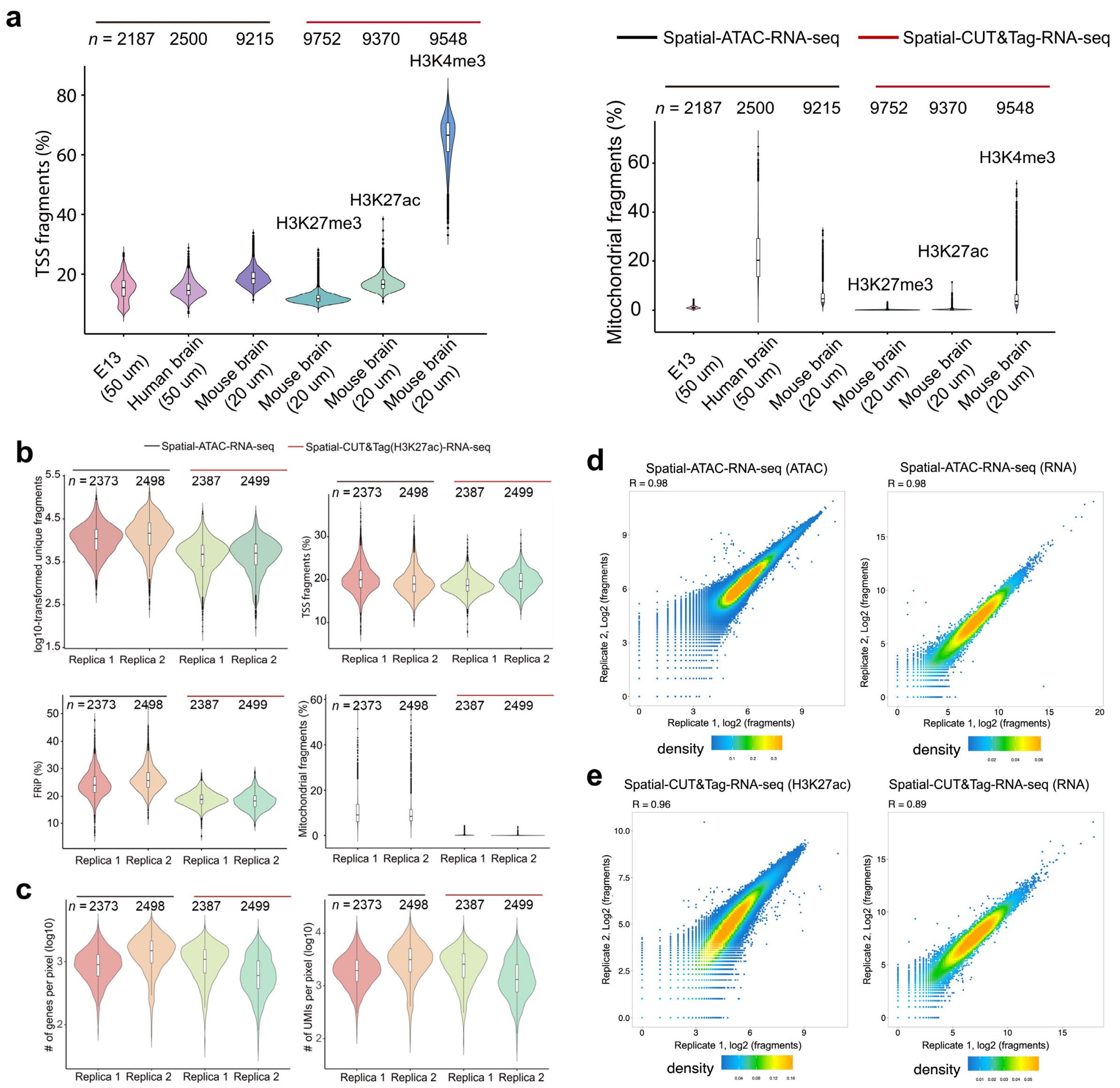

Supplementary Fig. 2 Quality control metrics for spatial-ATAC-RNA-seq and spatial-CUT&Tag-RNA-seq datasets. a, Comparison of TSS fragments and fraction of mitochondrial fragments in spatial-ATAC-RNA-seq and spatial-CUT&Tag-RNA-seq. **b,** Comparison of number of unique fragments, TSS fragments, fraction of reads in peaks (FRiP), and fraction of mitochondrial fragments between biological replicates for spatial-ATAC-RNA-seq and spatial-CUT&Tag(H3K27ac)-RNA-seq. **c,** Gene and UMI count distribution between biological replicates for spatial-ATAC-RNA-seq and spatial-CUT&Tag(H3K27ac)-RNA-seq. The box plots show the median (centre line), the first and third quartiles (box limits), and 1.5x the interquartile range (whiskers). **d,** The reproducibility of spatial-ATAC-RNA-seq between biological replicates on ATAC data (left) and RNA data (right) for P21 mouse brain. **e,** The reproducibility of spatial-CUT&Tag-RNA-seq between biological replicates on CUT&Tag data (left) and RNA data (right) for P21 mouse brain.

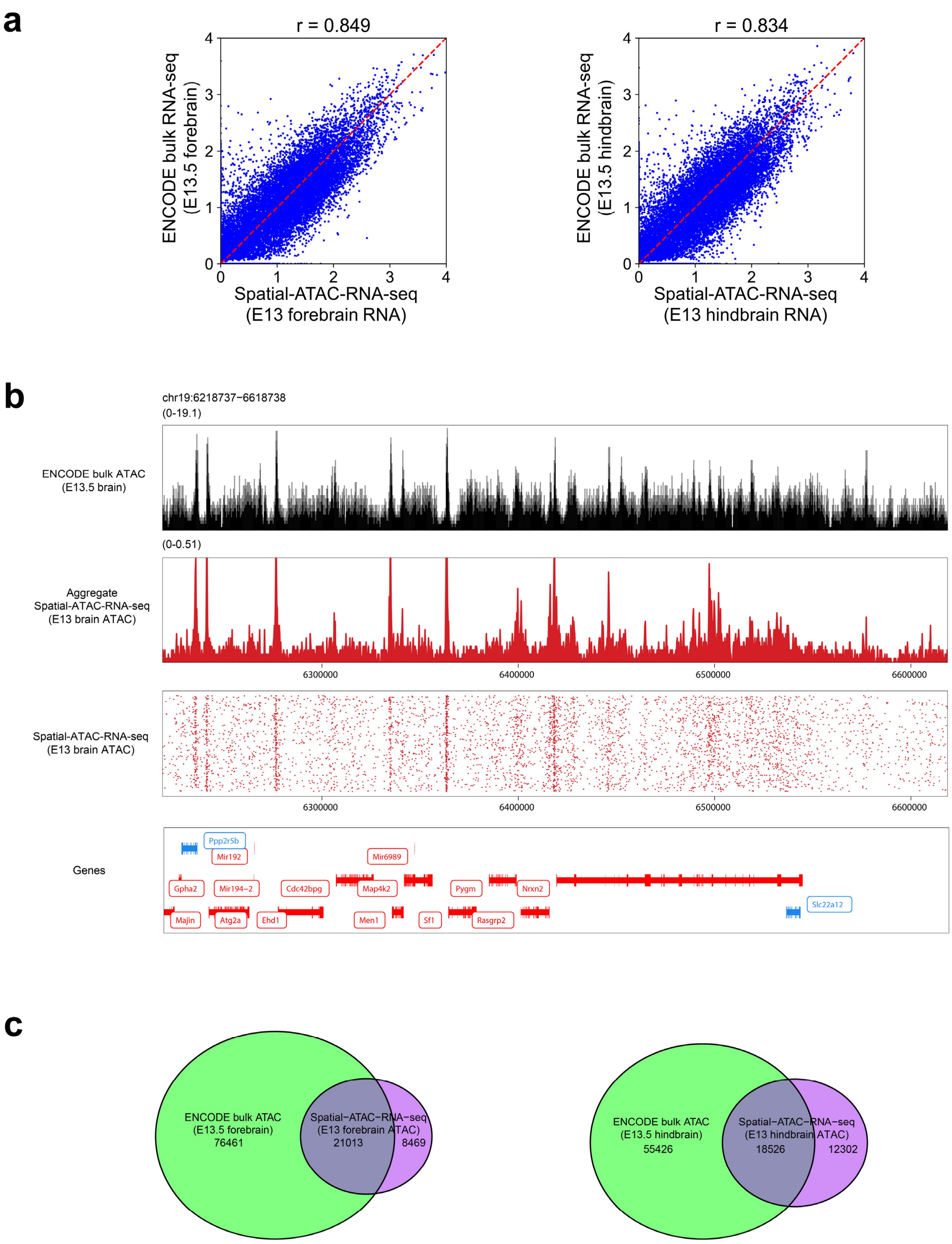

Supplementary Fig. 3 Benchmarking of data quality in spatial-ATAC-RNA-seq. a, Comparison of transcriptional profiles between RNA in spatial-ATAC-RNA-seq and the ENCODE RNA-Seq data in brain of mouse embryo. **b,** Aggregated spatial chromatin accessibility profiles in spatial-ATAC-RNA-seq recapitulated ENCODE ATAC-seq profiles in brain of mouse embryo. **c,** Venn diagrams showing the overlap of peaks from ATAC in spatial-ATAC-RNA-seq and ENCODE ATAC-seq in brain of mouse embryo.

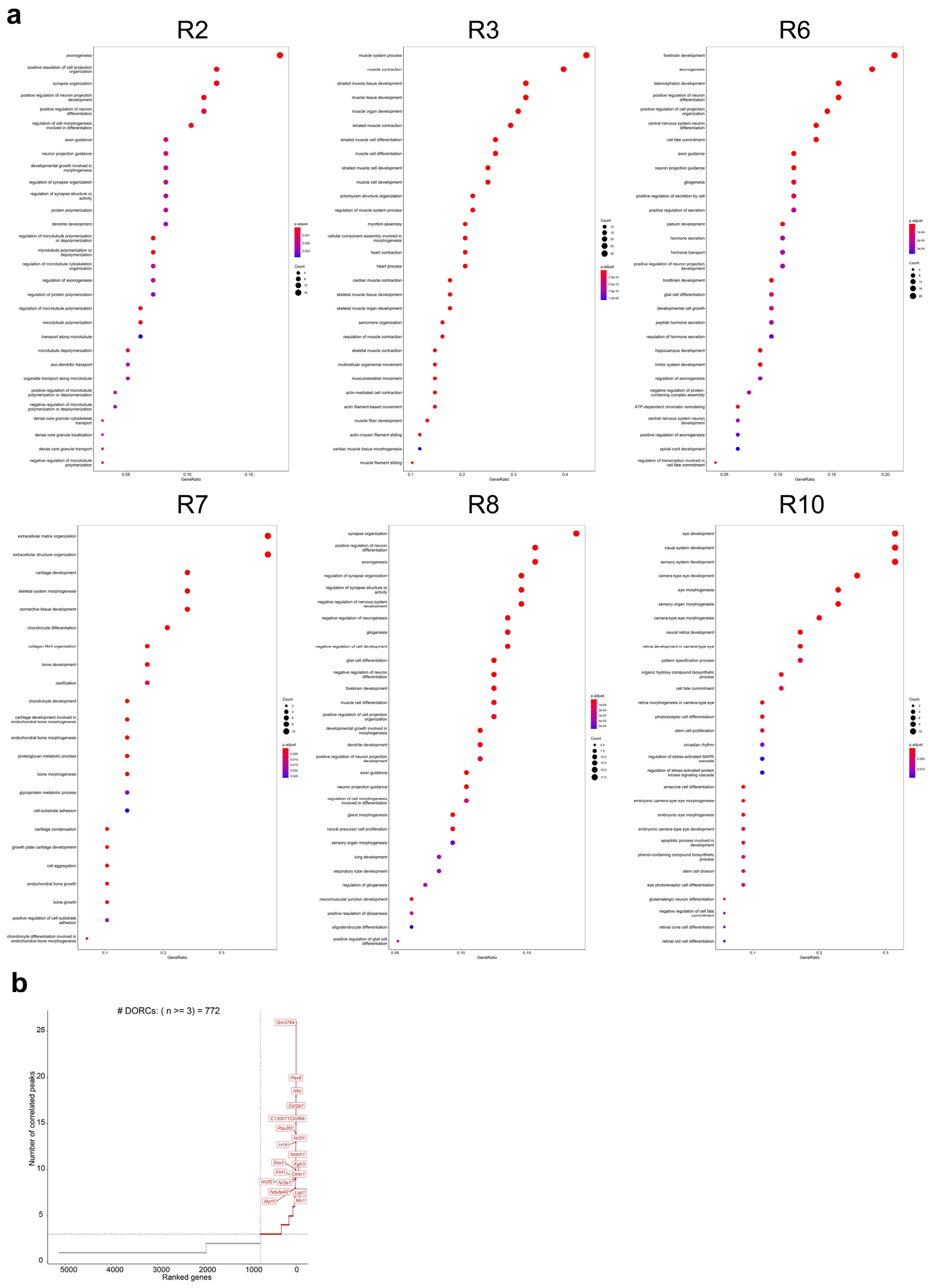

Supplementary Fig. 4 Further analysis of spatial-ATAC-RNA-seq for E13 mouse embryo. **a,** GO enrichment analysis in selected RNA clusters from spatial-ATAC-RNA-seq for E13 mouse embryo (One-sided version of Fisher's exact test, p-value was adjusted for multiple comparisons by Benjamini & Hochberg method). **b,** The number of significantly correlated peaks for each gene.

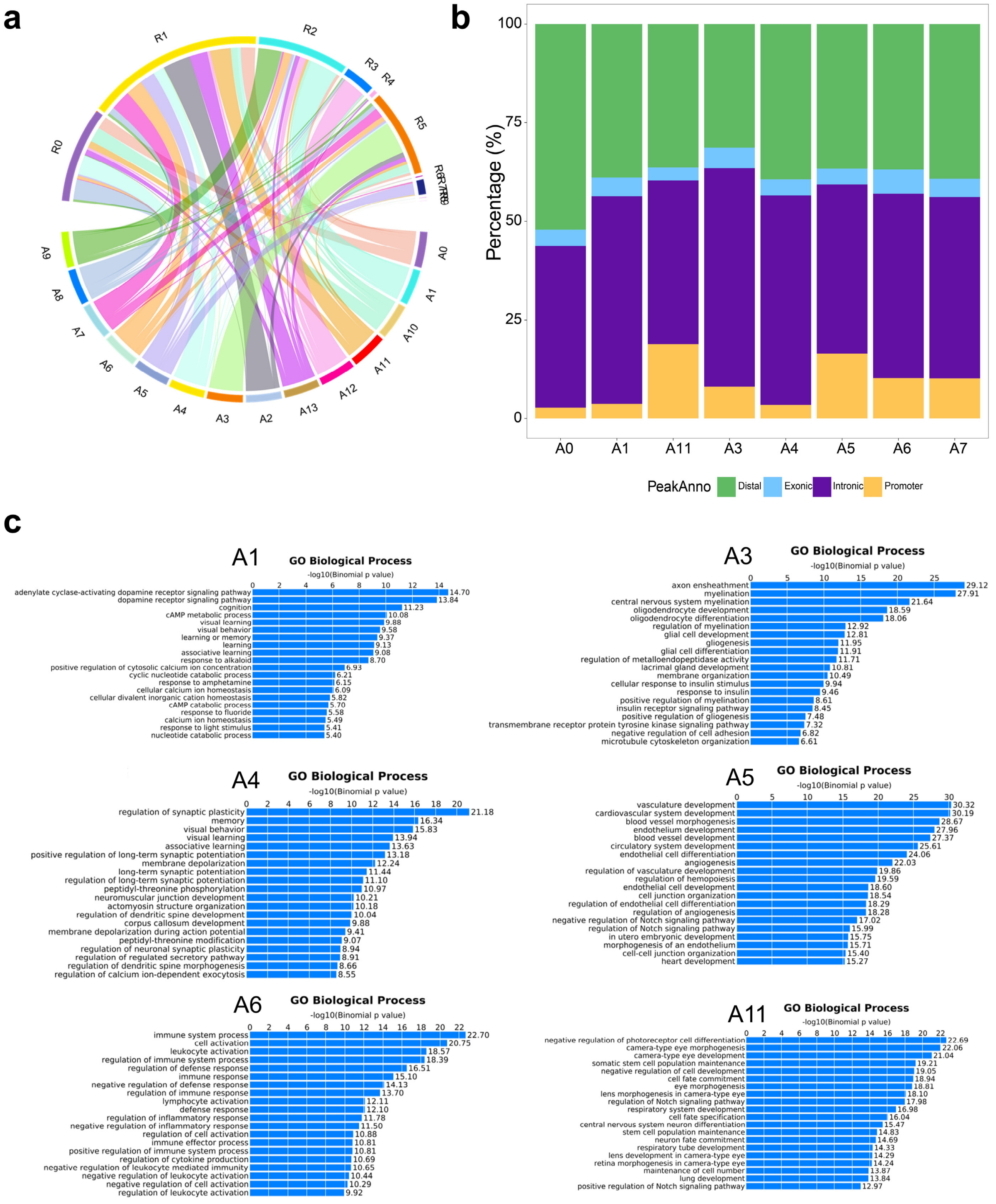

Supplementary Fig. 5 Further analysis of spatial-ATAC-RNA-seq for P22 mouse brain. a, Ribbon plot showing the relationship between ATAC and RNA clusters in spatial-ATAC-RNA-seq. **b,** Annotation of marker peaks in different clusters. **c,** GREAT enrichment analysis of marker peaks in different clusters (Binomial and hypergeometric tests).

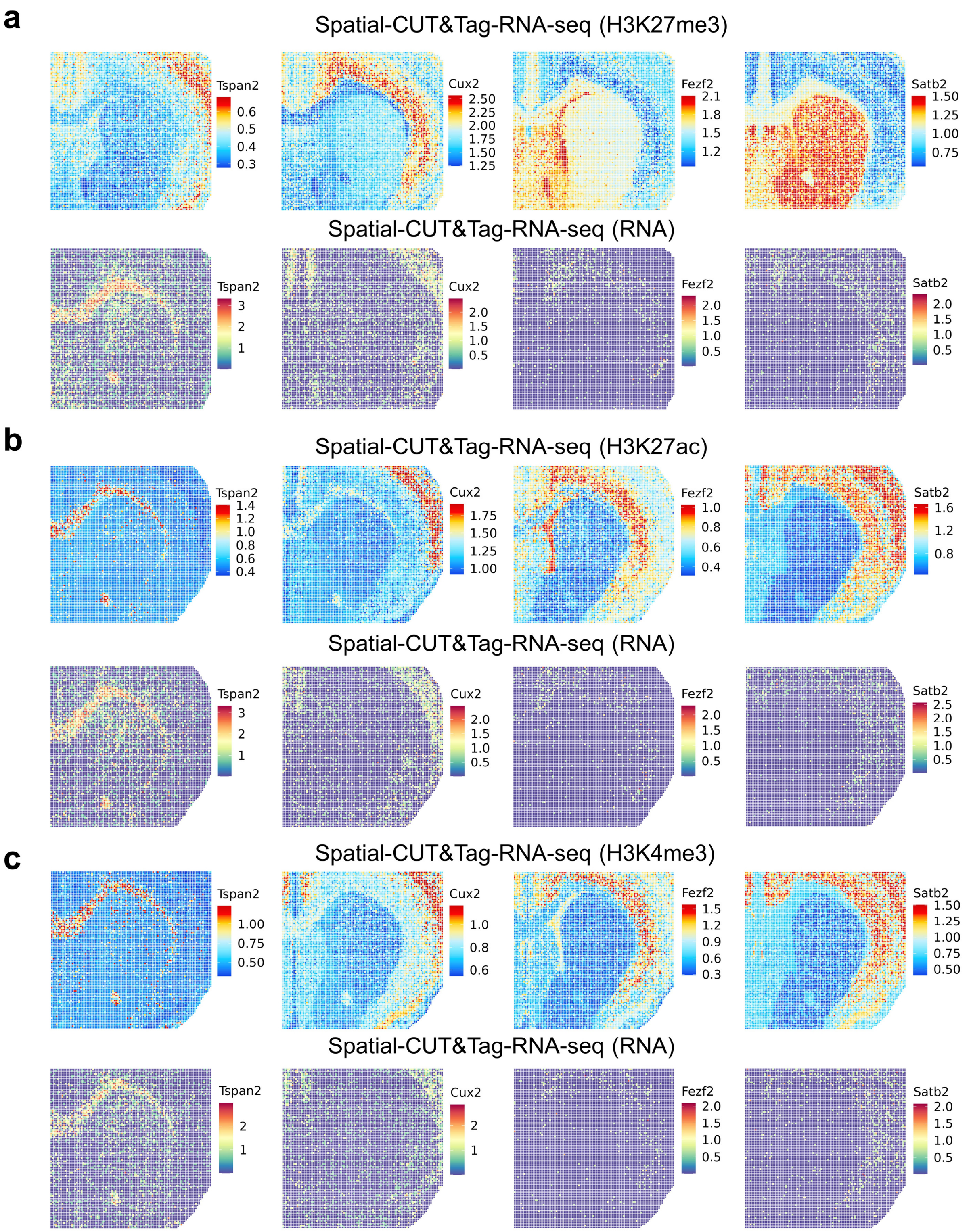

Supplementary Fig. 6 Spatial mapping of CSS, GAS, and gene expression of selected genes in spatial-CUT&Tag-RNA-seq for P22 mouse brain. a-c, Spatial mapping of CSS or GAS, and gene expression for selected marker genes in spatial-CUT&Tag(H3K27me3)-RNA-seq **(a)**, spatial-CUT&Tag(H3K27ac)-RNA-seq **(b)**, and spatial-CUT&Tag(H3K4me3)-RNA-seq **(c)**.

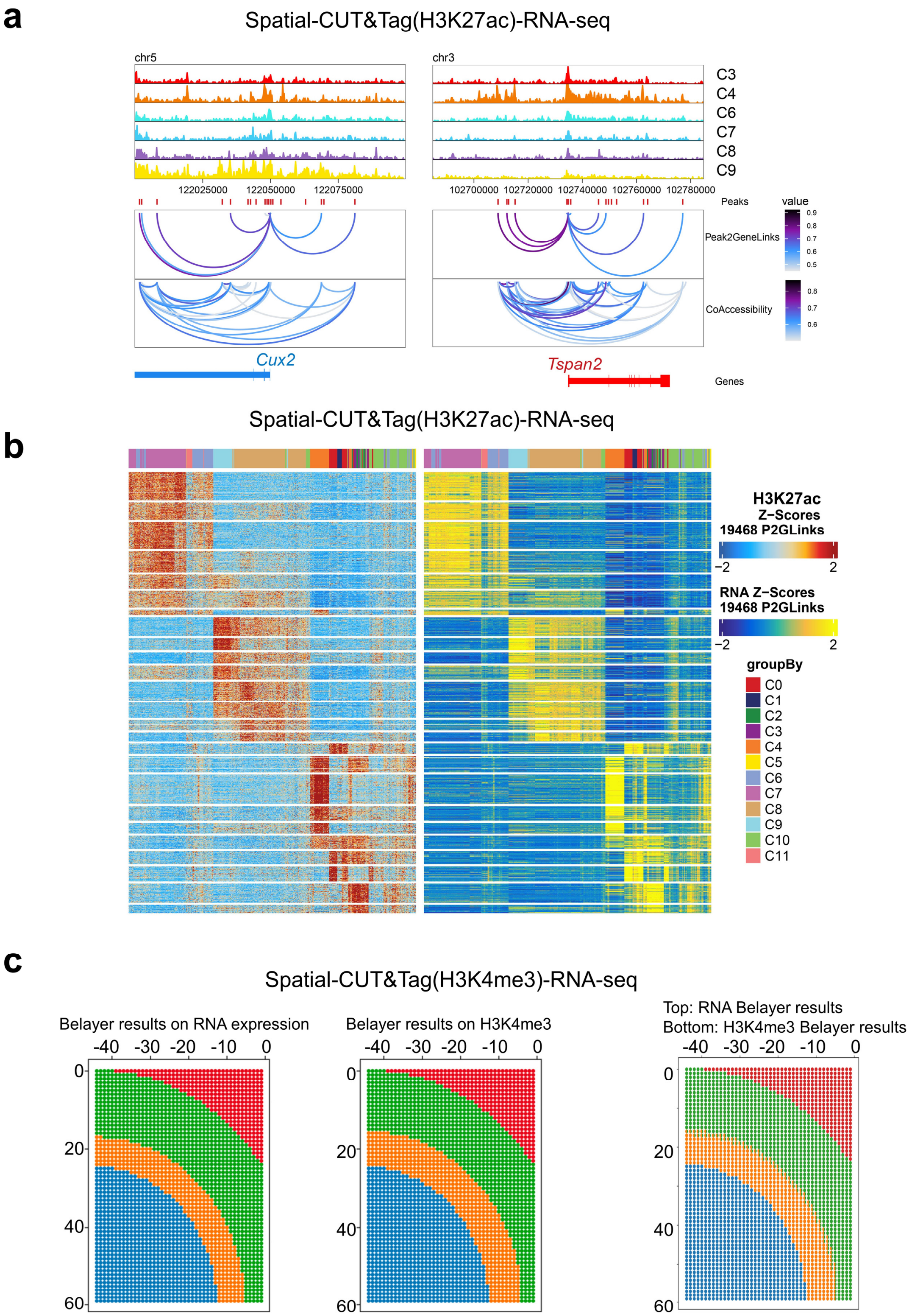

Supplementary Fig. 7 Further analysis for spatial-CUT&Tag-RNA-seq with P22 mouse brain. a, Genome track visualization of marker genes with peak-to-gene links for distal regulatory elements and peak co-accessibility. **b,** Heatmaps of peak-to-gene links in spatial-CUT&Tag(H3K27ac)-RNA-seq. **c,** The layers identified by Belayer from the top right mapping region of the P22 mouse brain. Evaluate the consistency between the two Belayer results (right) by adjusted rand index (ARI): ARI = 0.915.

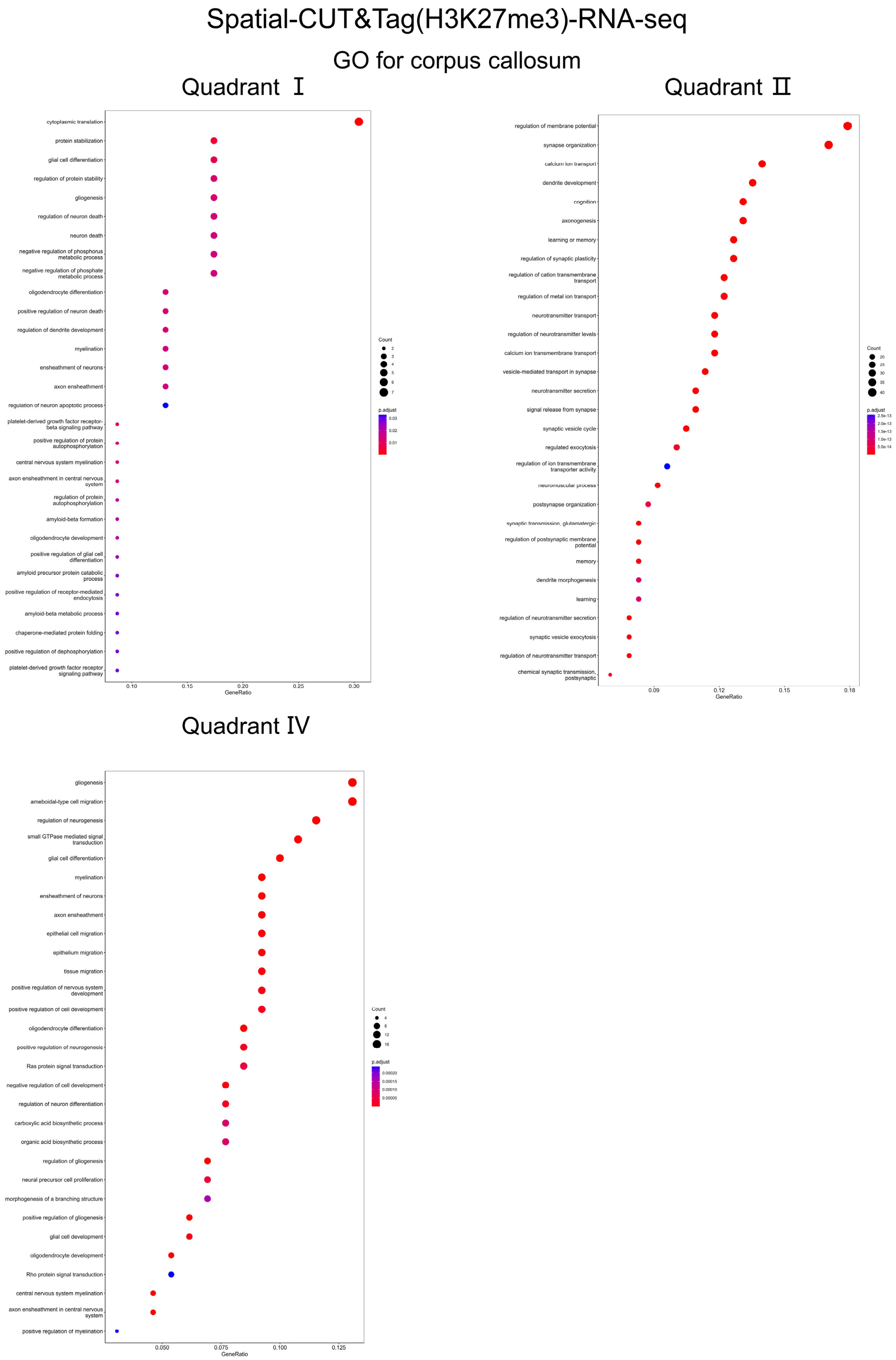

Supplementary Fig. 8 GO enrichment analysis of P22 mouse brain. GO enrichment analysis of genes from Fig. 4a (One-sided version of Fisher's exact test, p-value was adjusted for multiple comparisons by Benjamini & Hochberg method).

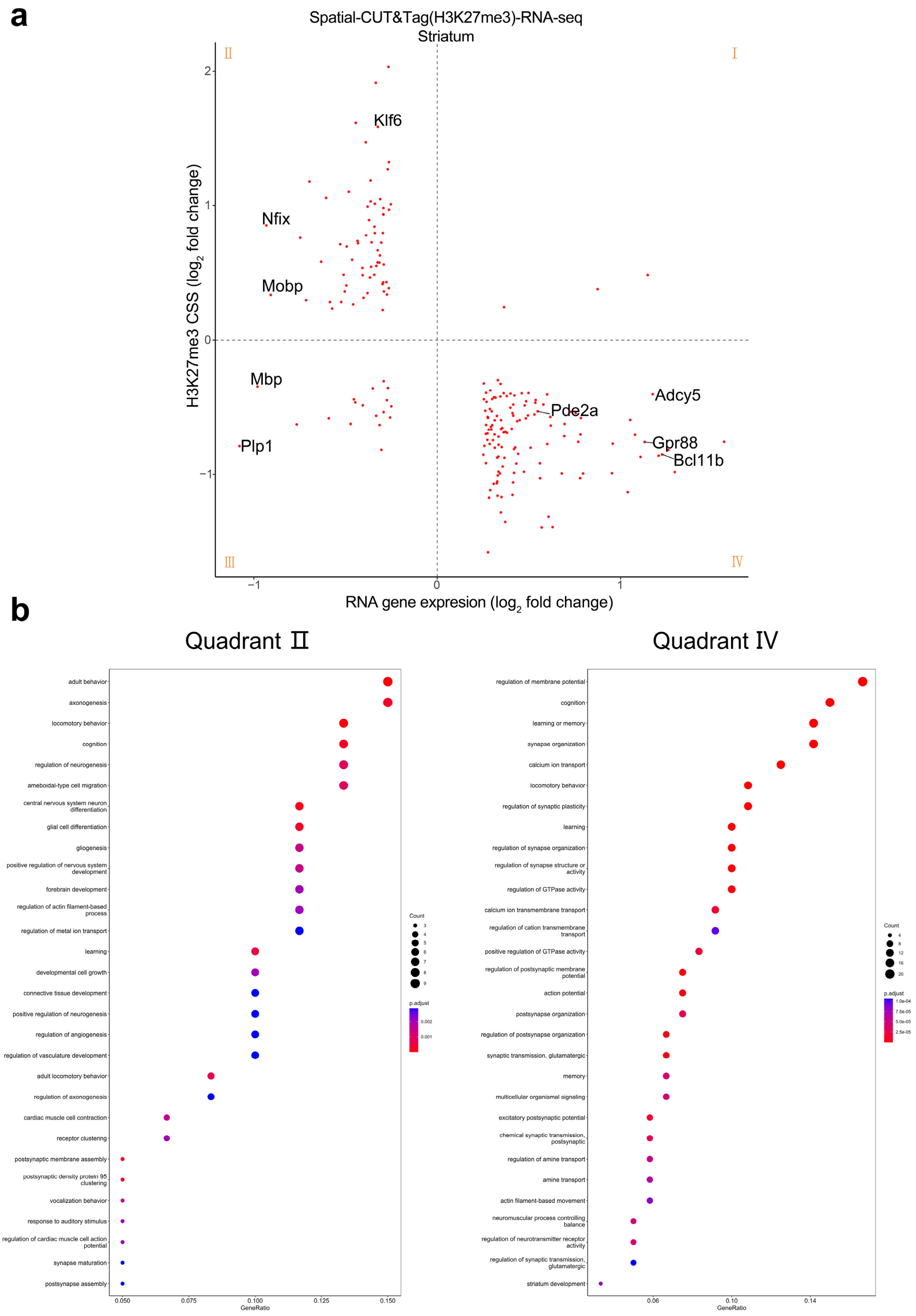

Supplementary Fig. 9 Further analysis for epigenetic regulation of gene expression with P22 mouse brain. a, Correlation analysis of H3K27me3 CSS and RNA gene expression in striatum. **b,** GO enrichment analysis of genes from (**a**) (One-sided version of Fisher's exact test, p-value was adjusted for multiple comparisons by Benjamini & Hochberg method).

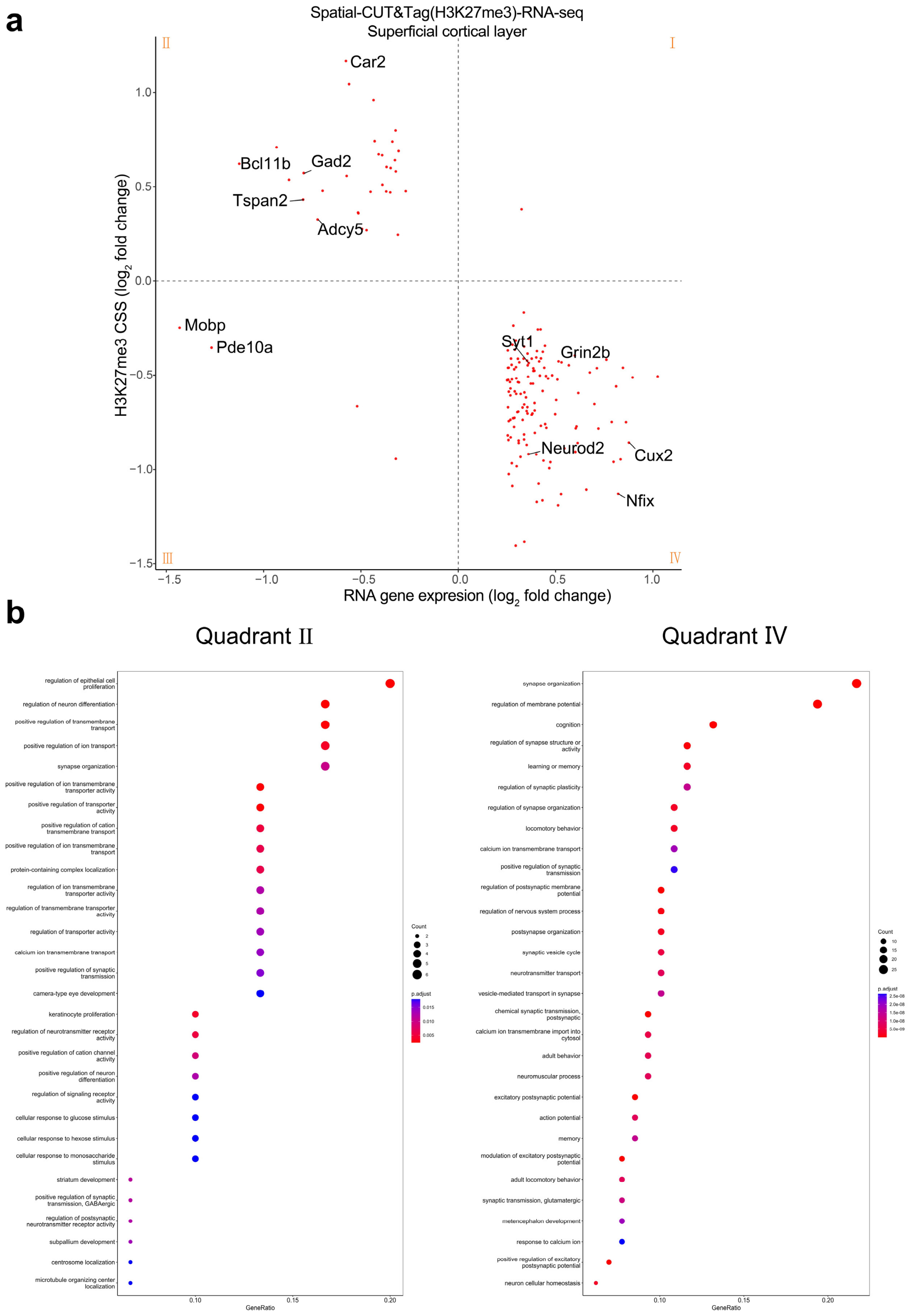

Supplementary Fig. 10 Further analysis for epigenetic regulation of gene expression with P22 mouse brain. a, Correlation analysis of H3K27me3 CSS and RNA gene expression in superficial cortical layer. **b,** GO enrichment analysis of genes from (**a**) (One-sided version of Fisher's exact test, p-value was adjusted for multiple comparisons by Benjamini & Hochberg method).

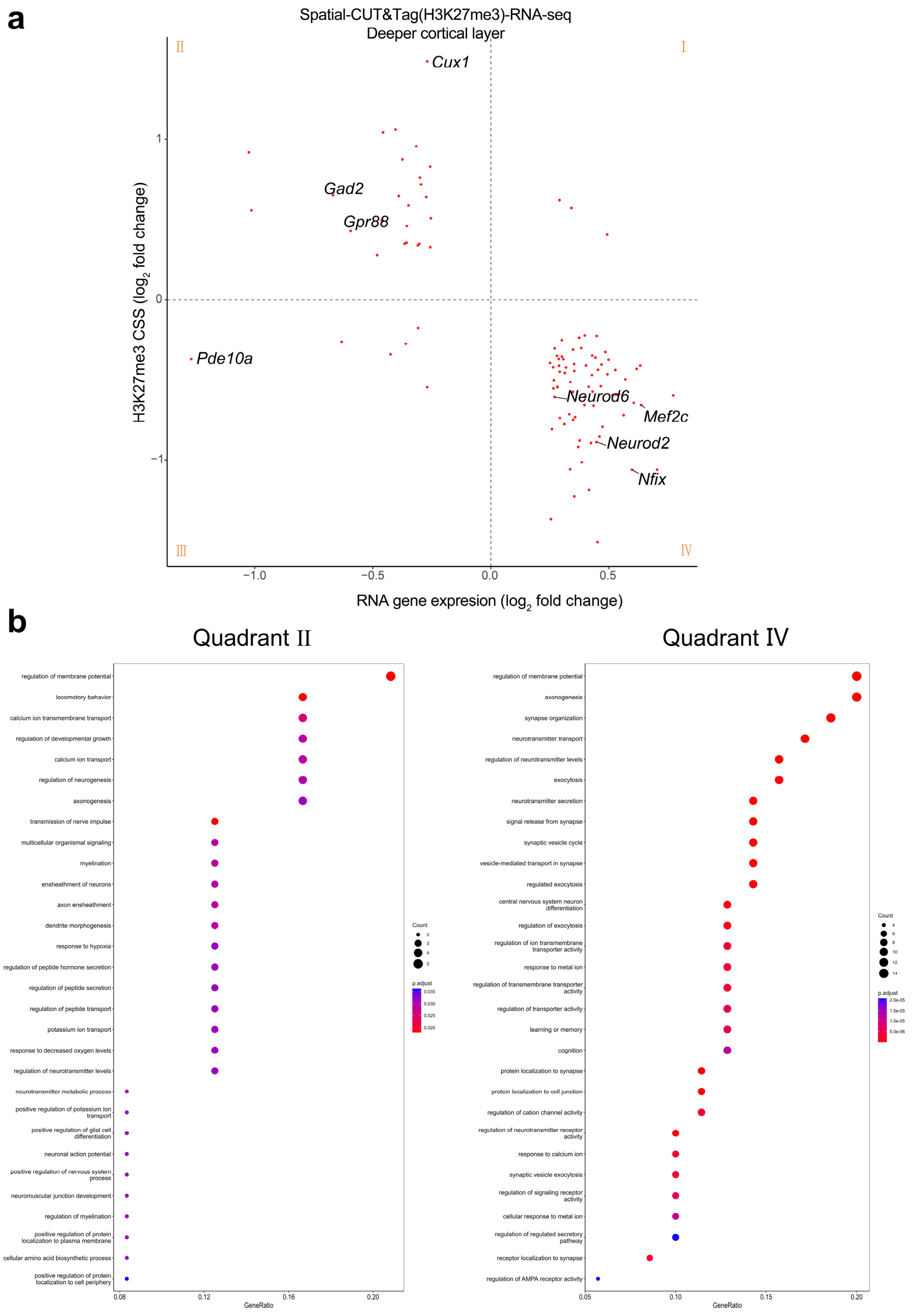

Supplementary Fig. 11 Further analysis for epigenetic regulation of gene expression with P22 mouse brain. a, Correlation analysis of H3K27me3 CSS and RNA gene expression in deeper cortical layer. **b,** GO enrichment analysis of genes from (**a**) (One-sided version of Fisher's exact test, p-value was adjusted for multiple comparisons by Benjamini & Hochberg method).

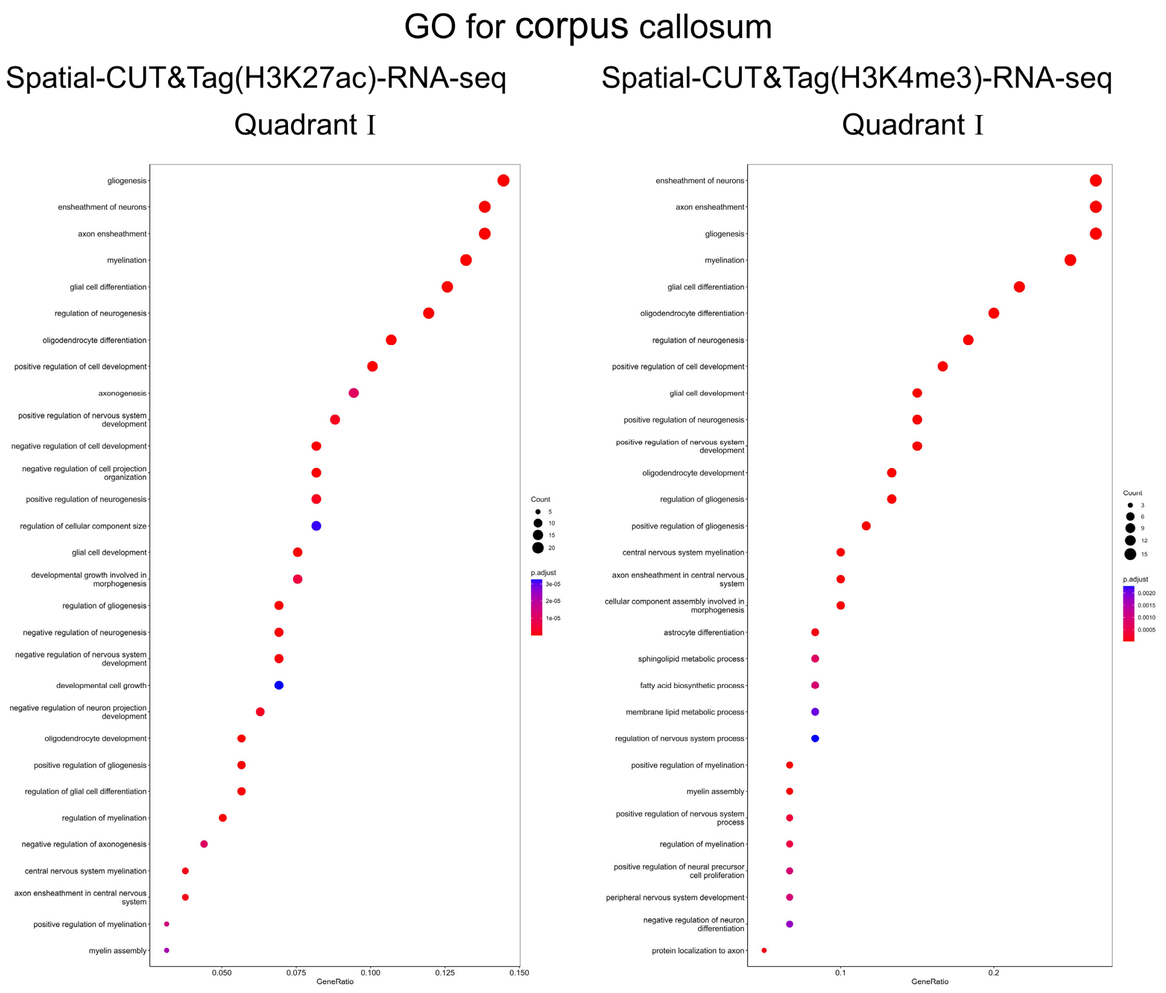

Supplementary Fig. 12 Further analysis for epigenetic regulation of gene expression with P22 mouse brain. GO enrichment analysis of genes from Fig. 4b,c (One-sided version of Fisher's exact test, p-value was adjusted for multiple comparisons by Benjamini & Hochberg method).

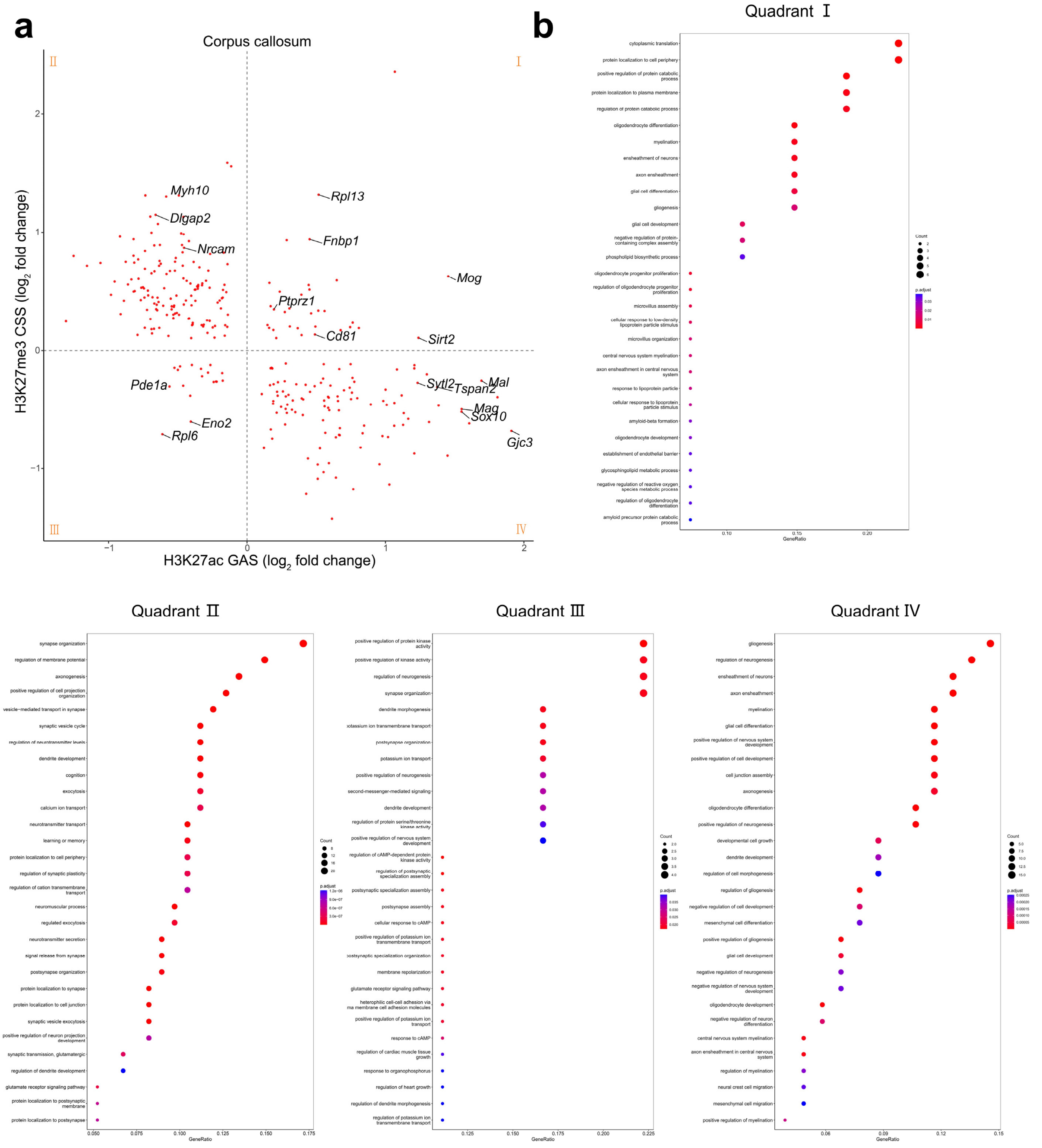

Supplementary Fig. 13 Further analysis for epigenetic regulation of gene expression with P22 mouse brain. a, Correlation analysis of H3K27me3 CSS and H3K27ac GAS in corpus callosum. **b,** GO enrichment analysis of genes from (**a**) (One-sided version of Fisher's exact test, p-value was adjusted for multiple comparisons by Benjamini & Hochberg method).

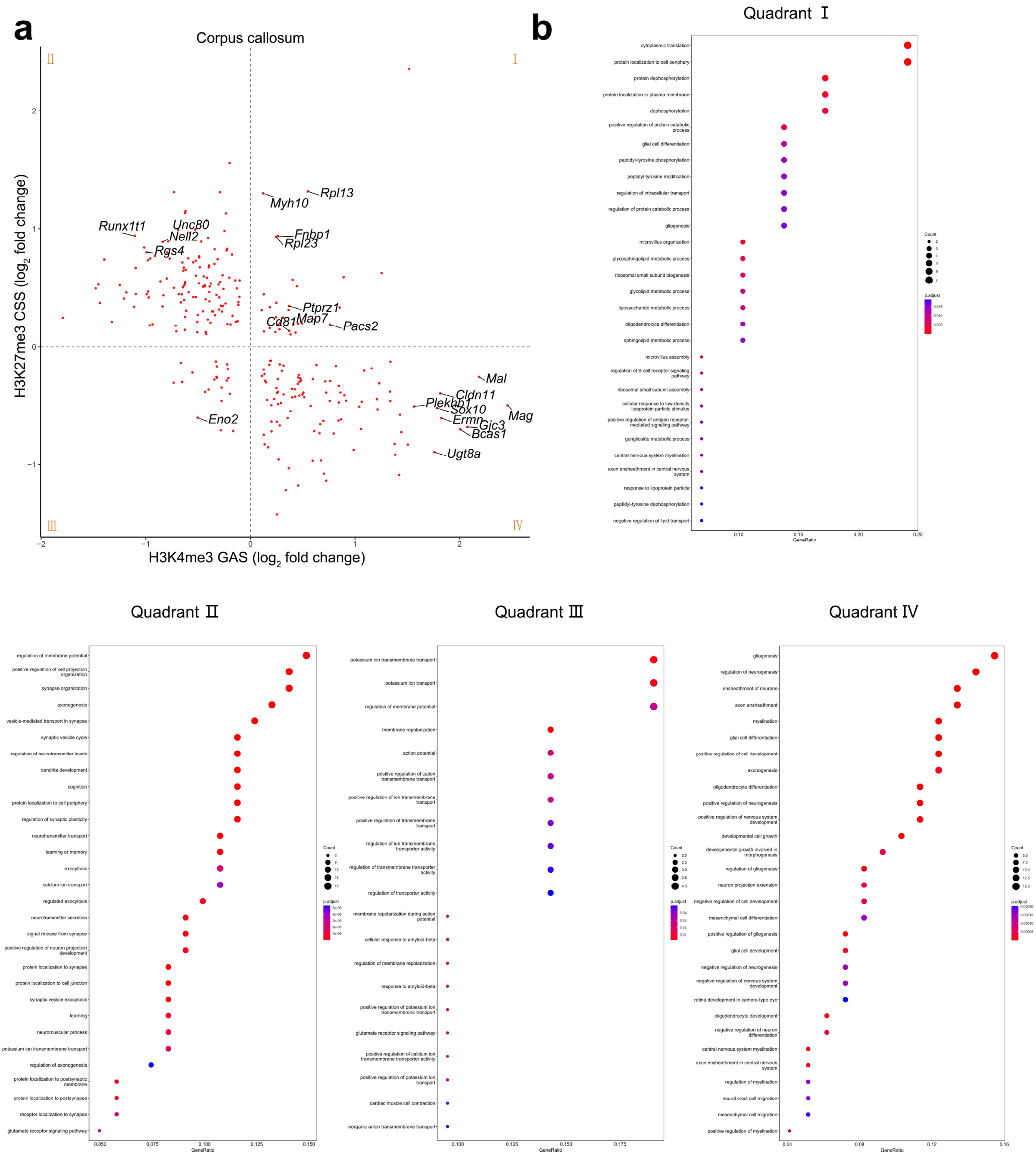

Supplementary Fig. 14 Further analysis for epigenetic regulation of gene expression with P22 mouse brain. a, Correlation analysis of H3K27me3 CSS and H3K4me3 GAS in corpus callosum. **b,** GO enrichment analysis of genes from (**a**) (One-sided version of Fisher's exact test, p-value was adjusted for multiple comparisons by Benjamini & Hochberg method).

Statistics and Reproducibility

Figure	Description	# of times repeated
Fig. 2b,3a	Nissl-stained image of P22 mouse brain	1
Fig. 3b,c	Nissl-stained image of P22 mouse brain	1
Fig. 5a, Extended Data Fig. 10a	Bright field image of human hippocampus	1
Extended Data Fig. 2a	H&E image of E13 mouse embryo	1
Extended Data Fig. 2d	Bright field image of E13 mouse embryo	1
Extended Data Fig. 4c,d	Bright field image of P22 mouse brain	1
Extended Data Fig. 6a,b,c	Bright field image of P21 mouse brain	1
Extended Data Fig. 6d,8d, Supplementary Fig. 1b	Nissl-stained image of P21 mouse brain	1
Extended Data Fig. 8a,b,c	Bright field image of P21 mouse brain	1

Supplementary Table 1. A list of cell type annotations in mouse brain.

Supplementary Table 2. Summary of metrics for ATAC and RNA in spatial-ATAC-RNA-seq for all the samples.

			E13 mouse embryo (50 barcodes, 50 µm pixel size)	P22 mouse brain (100 barcodes, 20 µm pixel size)	P21 mouse brain (replica 1, 50 barcodes, 20 µm pixel size)	P21 mouse brain (replica 2, 50 barcodes, 20 µm pixel size)	Human brain (50 barcodes, 50 µm pixel size)
Spatial-ATAC-RNA-seq	ATAC	Number of Unique fragments	18,079	14,284	10,857	14,385	9,898
		TSS fragments	16%	19%	20%	19%	15%
		FRiP	11%	26%	24%	26%	11%
		Mitochondrial fragments	0.96%	4.6%	9%	8.4%	20%
	RNA	Average number of genes per pixel	1,255	1,073	1,005	1,600	1,200
		Average number of UMIs per pixel	3,603	2,358	2,391	3,811	2,809
		Number of unique genes present	20,900	22,914	19,859	20,046	29,293
		Pixels on tissue	2,187	9,215	2,373	2,498	2,500

Supplementary Table 3. Summary of metrics for CUT&Tag and RNA in spatial-CUT&Tag-RNA-seq for all the samples.

			P22 mouse brain (H3K27me3) (100 barcodes, 20 µm pixel size)	P22 mouse brain (H3K27ac) (100 barcodes, 20 µm pixel size)	P22 mouse brain (H3K4me3) (100 barcodes, 20 µm pixel size)	P21 mouse brain (H3K27ac, replica 1) (50 barcodes, 20 µm pixel size)	P21 mouse brain (H3K27ac, replica 2) (50 barcodes, 20 µm pixel size)
Spatial-CUT&Tag-RNA-seq	CUT&Tag	Number of Unique fragments	10,644	10,002	2,507	4,756	5,022
		TSS fragments	12%	17%	67%	19%	20%
		FRiP	12%	21%	54%	19%	18%
		Mitochondrial fragments	0.2%	0.3%	3.6%	0.1%	0.02%
	RNA	Average number of genes per pixel	2,011	1,513	1,329	1,145	752
		Average number of UMIs per pixel	4,734	3,580	2,885	2,938	1,890
		Number of unique genes present	25,881	23,415	22,731	19,831	18,718
		Pixels on tissue	9,752	9,370	9,548	2,387	2,499

Supplementary Table 4. DNA oligos used for PCR and preparation of sequencing library.

RT primer	/5Phos/CATCGGCGTACGACTNNNNNNNNNN/iBiodT/TTTTTTTTTTTTTTTVN
Ligation linker 1	AGTCGTACGCCGATGCGAAACATCGGCCAC
Ligation linker 2	CGAATGCTCTGGCCTCTCAAGCACGTGGAT
PCR Primer 1	CAAGCGTTGGCTTCTCGCATCT
PCR Primer 2	AAGCAGTGGTATCAACGCAGAGT
N501	AATGATACGGCGACCACCGAGATCTACACTAGATCGCTCGTCGGCAGCGTCAGATGTGTATAAGAGACAG
N701	CAAGCAGAAGACGGCATACGAGATTCGCCTTAGTCTCGTGGGCTCGGAGATGTGTATAAGAGACAGCAAGCGTTGGCTTCTCGCATCT
N702	CAAGCAGAAGACGGCATACGAGATCTAGTACGGTCTCGTGGGCTCGGAGATGTGTATAAGAGACAGCAAGCGTTGGCTTCTCGCATCT
N703	CAAGCAGAAGACGGCATACGAGATTTCTGCCTGTCTCGTGGGCTCGGAGATGTGTATAAGAGACAGCAAGCGTTGGCTTCTCGCATCT
N704	CAAGCAGAAGACGGCATACGAGATGCTCAGGAGTCTCGTGGGCTCGGAGATGTGTATAAGAGACAGCAAGCGTTGGCTTCTCGCATCT
N705	CAAGCAGAAGACGGCATACGAGATAGGAGTCCGTCTCGTGGGCTCGGAGATGTGTATAAGAGACAGCAAGCGTTGGCTTCTCGCATCT
N706	CAAGCAGAAGACGGCATACGAGATCATGCCTAGTCTCGTGGGCTCGGAGATGTGTATAAGAGACAGCAAGCGTTGGCTTCTCGCATCT
N707	CAAGCAGAAGACGGCATACGAGATGTAGAGAGGTCTCGTGGGCTCGGAGATGTGTATAAGAGACAGCAAGCGTTGGCTTCTCGCATCT

Supplementary Table 5. DNA barcode A sequences.

Barcode A	Sequence
Barcode A-1	/5Phos/AGGCCAGAGCATTCGAACGTGATGTGGCCGATGTTTCG
Barcode A-2	/5Phos/AGGCCAGAGCATTCGAAACATCGGTGGCCGATGTTTCG
Barcode A-3	/5Phos/AGGCCAGAGCATTCGATGCCTAAGTGGCCGATGTTTCG
Barcode A-4	/5Phos/AGGCCAGAGCATTCGAGTGGTCAGTGGCCGATGTTTCG
Barcode A-5	/5Phos/AGGCCAGAGCATTCGACCACTGTGTGGCCGATGTTTCG
Barcode A-6	/5Phos/AGGCCAGAGCATTCGACATTGGCGTGGCCGATGTTTCG
Barcode A-7	/5Phos/AGGCCAGAGCATTCGCAGATCTGGTGGCCGATGTTTCG
Barcode A-8	/5Phos/AGGCCAGAGCATTCGCATCAAGTGTGGCCGATGTTTCG
Barcode A-9	/5Phos/AGGCCAGAGCATTCGCGCTGATCGTGGCCGATGTTTCG
Barcode A-10	/5Phos/AGGCCAGAGCATTCGACAAGCTAGTGGCCGATGTTTCG
Barcode A-11	/5Phos/AGGCCAGAGCATTCGCTGTAGCCGTGGCCGATGTTTCG
Barcode A-12	/5Phos/AGGCCAGAGCATTCGAGTACAAGGTGGCCGATGTTTCG
Barcode A-13	/5Phos/AGGCCAGAGCATTCGAACAACCAGTGGCCGATGTTTCG
Barcode A-14	/5Phos/AGGCCAGAGCATTCGAACCGAGAGTGGCCGATGTTTCG
Barcode A-15	/5Phos/AGGCCAGAGCATTCGAACGCTTAGTGGCCGATGTTTCG
Barcode A-16	/5Phos/AGGCCAGAGCATTCGAAGACGGAGTGGCCGATGTTTCG
Barcode A-17	/5Phos/AGGCCAGAGCATTCGAAGGTACAGTGGCCGATGTTTCG
Barcode A-18	/5Phos/AGGCCAGAGCATTCGACACAGAAGTGGCCGATGTTTCG
Barcode A-19	/5Phos/AGGCCAGAGCATTCGACAGCAGAGTGGCCGATGTTTCG
Barcode A-20	/5Phos/AGGCCAGAGCATTCGACCTCCAAGTGGCCGATGTTTCG
Barcode A-21	/5Phos/AGGCCAGAGCATTCGACGCTCGAGTGGCCGATGTTTCG
Barcode A-22	/5Phos/AGGCCAGAGCATTCGACGTATCAGTGGCCGATGTTTCG
Barcode A-23	/5Phos/AGGCCAGAGCATTCGACTATGCAGTGGCCGATGTTTCG
Barcode A-24	/5Phos/AGGCCAGAGCATTCGAGAGTCAAGTGGCCGATGTTTCG
Barcode A-25	/5Phos/AGGCCAGAGCATTCGAGATCGCAGTGGCCGATGTTTCG
Barcode A-26	/5Phos/AGGCCAGAGCATTCGAGCAGGAAGTGGCCGATGTTTCG
Barcode A-27	/5Phos/AGGCCAGAGCATTCGAGTCACTAGTGGCCGATGTTTCG
Barcode A-28	/5Phos/AGGCCAGAGCATTCGATCCTGTAGTGGCCGATGTTTCG
Barcode A-29	/5Phos/AGGCCAGAGCATTCGATTGAGGAGTGGCCGATGTTTCG
Barcode A-30	/5Phos/AGGCCAGAGCATTCGCAACCACAGTGGCCGATGTTTCG
Barcode A-31	/5Phos/AGGCCAGAGCATTCGGACTAGTAGTGGCCGATGTTTCG
Barcode A-32	/5Phos/AGGCCAGAGCATTCGCAATGGAAGTGGCCGATGTTTCG
Barcode A-33	/5Phos/AGGCCAGAGCATTCGCACTTCGAGTGGCCGATGTTTCG
Barcode A-34	/5Phos/AGGCCAGAGCATTCGCAGCGTTAGTGGCCGATGTTTCG
Barcode A-35	/5Phos/AGGCCAGAGCATTCGCATACCAAGTGGCCGATGTTTCG
Barcode A-36	/5Phos/AGGCCAGAGCATTCGCCAGTTCAGTGGCCGATGTTTCG
Barcode A-37	/5Phos/AGGCCAGAGCATTCGCCGAAGTAGTGGCCGATGTTTCG
Barcode A-38	/5Phos/AGGCCAGAGCATTCGCCGTGAGAGTGGCCGATGTTTCG
Barcode A-39	/5Phos/AGGCCAGAGCATTCGCCTCCTGAGTGGCCGATGTTTCG
Barcode A-40	/5Phos/AGGCCAGAGCATTCGCGAACTTAGTGGCCGATGTTTCG
Barcode A-41	/5Phos/AGGCCAGAGCATTCGCGACTGGAGTGGCCGATGTTTCG

Barcode A-42	/5Phos/AGGCCAGAGCATTCGCGCATACAGTGGCCGATGTTTCG
Barcode A-43	/5Phos/AGGCCAGAGCATTCGCTCAATGAGTGGCCGATGTTTCG
Barcode A-44	/5Phos/AGGCCAGAGCATTCGCTGAGCCAGTGGCCGATGTTTCG
Barcode A-45	/5Phos/AGGCCAGAGCATTCGCTGGCATAGTGGCCGATGTTTCG
Barcode A-46	/5Phos/AGGCCAGAGCATTCGGAATCTGAGTGGCCGATGTTTCG
Barcode A-47	/5Phos/AGGCCAGAGCATTCGCAAGACTAGTGGCCGATGTTTCG
Barcode A-48	/5Phos/AGGCCAGAGCATTCGGAGCTGAAGTGGCCGATGTTTCG
Barcode A-49	/5Phos/AGGCCAGAGCATTCGGATAGACAGTGGCCGATGTTTCG
Barcode A-50	/5Phos/AGGCCAGAGCATTCGGCCACATAGTGGCCGATGTTTCG
Barcode A-51	/5Phos/AGGCCAGAGCATTCGGCGAGTAAGTGGCCGATGTTTCG
Barcode A-52	/5Phos/AGGCCAGAGCATTCGGCTAACGAGTGGCCGATGTTTCG
Barcode A-53	/5Phos/AGGCCAGAGCATTCGGCTCGGTAGTGGCCGATGTTTCG
Barcode A-54	/5Phos/AGGCCAGAGCATTCGGGAGAACAGTGGCCGATGTTTCG
Barcode A-55	/5Phos/AGGCCAGAGCATTCGGGTGCGAAGTGGCCGATGTTTCG
Barcode A-56	/5Phos/AGGCCAGAGCATTCGGTACGCAAGTGGCCGATGTTTCG
Barcode A-57	/5Phos/AGGCCAGAGCATTCGGTCGTAGAGTGGCCGATGTTTCG
Barcode A-58	/5Phos/AGGCCAGAGCATTCGGTCTGTCAGTGGCCGATGTTTCG
Barcode A-59	/5Phos/AGGCCAGAGCATTCGGTGTTCTAGTGGCCGATGTTTCG
Barcode A-60	/5Phos/AGGCCAGAGCATTCGTAGGATGAGTGGCCGATGTTTCG
Barcode A-61	/5Phos/AGGCCAGAGCATTCGTATCAGCAGTGGCCGATGTTTCG
Barcode A-62	/5Phos/AGGCCAGAGCATTCGTCCGTCTAGTGGCCGATGTTTCG
Barcode A-63	/5Phos/AGGCCAGAGCATTCGTCTTCACAGTGGCCGATGTTTCG
Barcode A-64	/5Phos/AGGCCAGAGCATTCGTGAAGAGAGTGGCCGATGTTTCG
Barcode A-65	/5Phos/AGGCCAGAGCATTCGTGGAACAAGTGGCCGATGTTTCG
Barcode A-66	/5Phos/AGGCCAGAGCATTCGTGGCTTCAGTGGCCGATGTTTCG
Barcode A-67	/5Phos/AGGCCAGAGCATTCGTGGTGGTAGTGGCCGATGTTTCG
Barcode A-68	/5Phos/AGGCCAGAGCATTCGTTCACGCAGTGGCCGATGTTTCG
Barcode A-69	/5Phos/AGGCCAGAGCATTCGAACTCACCGTGGCCGATGTTTCG
Barcode A-70	/5Phos/AGGCCAGAGCATTCGAAGAGATCGTGGCCGATGTTTCG
Barcode A-71	/5Phos/AGGCCAGAGCATTCGAAGGACACGTGGCCGATGTTTCG
Barcode A-72	/5Phos/AGGCCAGAGCATTCGAATCCGTCGTGGCCGATGTTTCG
Barcode A-73	/5Phos/AGGCCAGAGCATTCGAATGTTGCGTGGCCGATGTTTCG
Barcode A-74	/5Phos/AGGCCAGAGCATTCGACACGACCGTGGCCGATGTTTCG
Barcode A-75	/5Phos/AGGCCAGAGCATTCGACAGATTCGTGGCCGATGTTTCG
Barcode A-76	/5Phos/AGGCCAGAGCATTCGAGATGTACGTGGCCGATGTTTCG
Barcode A-77	/5Phos/AGGCCAGAGCATTCGAGCACCTCGTGGCCGATGTTTCG
Barcode A-78	/5Phos/AGGCCAGAGCATTCGAGCCATGCGTGGCCGATGTTTCG
Barcode A-79	/5Phos/AGGCCAGAGCATTCGAGGCTAACGTGGCCGATGTTTCG
Barcode A-80	/5Phos/AGGCCAGAGCATTCGATAGCGACGTGGCCGATGTTTCG
Barcode A-81	/5Phos/AGGCCAGAGCATTCGATCATTCCGTGGCCGATGTTTCG
Barcode A-82	/5Phos/AGGCCAGAGCATTCGATTGGCTCGTGGCCGATGTTTCG
Barcode A-83	/5Phos/AGGCCAGAGCATTCGCAAGGAGCGTGGCCGATGTTTCG
Barcode A-84	/5Phos/AGGCCAGAGCATTCGCACCTTACGTGGCCGATGTTTCG

Barcode A-85	/5Phos/AGGCCAGAGCATTCGCCATCCTCGTGGCCGATGTTTCG
Barcode A-86	/5Phos/AGGCCAGAGCATTCGCCGACAACGTGGCCGATGTTTCG
Barcode A-87	/5Phos/AGGCCAGAGCATTCGCCTAATCCGTGGCCGATGTTTCG
Barcode A-88	/5Phos/AGGCCAGAGCATTCGCCTCTATCGTGGCCGATGTTTCG
Barcode A-89	/5Phos/AGGCCAGAGCATTCGCGACACACGTGGCCGATGTTTCG
Barcode A-90	/5Phos/AGGCCAGAGCATTCGCGGATTGCGTGGCCGATGTTTCG
Barcode A-91	/5Phos/AGGCCAGAGCATTCGCTAAGGTCGTGGCCGATGTTTCG
Barcode A-92	/5Phos/AGGCCAGAGCATTCGGAACAGGCGTGGCCGATGTTTCG
Barcode A-93	/5Phos/AGGCCAGAGCATTCGGACAGTGCGTGGCCGATGTTTCG
Barcode A-94	/5Phos/AGGCCAGAGCATTCGGAGTTAGCGTGGCCGATGTTTCG
Barcode A-95	/5Phos/AGGCCAGAGCATTCGGATGAATCGTGGCCGATGTTTCG
Barcode A-96	/5Phos/AGGCCAGAGCATTCGGCCAAGACGTGGCCGATGTTTCG
Barcode A-97	/5Phos/AGGCCAGAGCATTCGCGGAAGAAGTGGCCGATGTTTCG
Barcode A-98	/5Phos/AGGCCAGAGCATTCGGTGACAAGGTGGCCGATGTTTCG
Barcode A-99	/5Phos/AGGCCAGAGCATTCGGAACCAGAGTGGCCGATGTTTCG
Barcode A-100	/5Phos/AGGCCAGAGCATTCGTTGCTGGAGTGGCCGATGTTTCG

Supplementary Table 6. DNA barcode B sequences.

Barcode B	Sequence
Barcode B-1	CAAGCGTTGGCTTCTCGCATCTAACGTGATATCCACGTGCTTGAG
Barcode B-2	CAAGCGTTGGCTTCTCGCATCTAAACATCGATCCACGTGCTTGAG
Barcode B-3	CAAGCGTTGGCTTCTCGCATCTATGCCTAAATCCACGTGCTTGAG
Barcode B-4	CAAGCGTTGGCTTCTCGCATCTAGTGGTCAATCCACGTGCTTGAG
Barcode B-5	CAAGCGTTGGCTTCTCGCATCTACCACTGTATCCACGTGCTTGAG
Barcode B-6	CAAGCGTTGGCTTCTCGCATCTACATTGGCATCCACGTGCTTGAG
Barcode B-7	CAAGCGTTGGCTTCTCGCATCTCAGATCTGATCCACGTGCTTGAG
Barcode B-8	CAAGCGTTGGCTTCTCGCATCTCATCAAGTATCCACGTGCTTGAG
Barcode B-9	CAAGCGTTGGCTTCTCGCATCTCGCTGATCATCCACGTGCTTGAG
Barcode B-10	CAAGCGTTGGCTTCTCGCATCTACAAGCTAATCCACGTGCTTGAG
Barcode B-11	CAAGCGTTGGCTTCTCGCATCTCTGTAGCCATCCACGTGCTTGAG
Barcode B-12	CAAGCGTTGGCTTCTCGCATCTAGTACAAGATCCACGTGCTTGAG
Barcode B-13	CAAGCGTTGGCTTCTCGCATCTAACAACCAATCCACGTGCTTGAG
Barcode B-14	CAAGCGTTGGCTTCTCGCATCTAACCGAGAATCCACGTGCTTGAG
Barcode B-15	CAAGCGTTGGCTTCTCGCATCTAACGCTTAATCCACGTGCTTGAG
Barcode B-16	CAAGCGTTGGCTTCTCGCATCTAAGACGGAATCCACGTGCTTGAG
Barcode B-17	CAAGCGTTGGCTTCTCGCATCTAAGGTACAATCCACGTGCTTGAG
Barcode B-18	CAAGCGTTGGCTTCTCGCATCTACACAGAAATCCACGTGCTTGAG
Barcode B-19	CAAGCGTTGGCTTCTCGCATCTACAGCAGAATCCACGTGCTTGAG
Barcode B-20	CAAGCGTTGGCTTCTCGCATCTACCTCCAAATCCACGTGCTTGAG
Barcode B-21	CAAGCGTTGGCTTCTCGCATCTACGCTCGAATCCACGTGCTTGAG
Barcode B-22	CAAGCGTTGGCTTCTCGCATCTACGTATCAATCCACGTGCTTGAG
Barcode B-23	CAAGCGTTGGCTTCTCGCATCTACTATGCAATCCACGTGCTTGAG
Barcode B-24	CAAGCGTTGGCTTCTCGCATCTAGAGTCAAATCCACGTGCTTGAG
Barcode B-25	CAAGCGTTGGCTTCTCGCATCTAGATCGCAATCCACGTGCTTGAG
Barcode B-26	CAAGCGTTGGCTTCTCGCATCTAGCAGGAAATCCACGTGCTTGAG
Barcode B-27	CAAGCGTTGGCTTCTCGCATCTAGTCACTAATCCACGTGCTTGAG
Barcode B-28	CAAGCGTTGGCTTCTCGCATCTATCCTGTAATCCACGTGCTTGAG
Barcode B-29	CAAGCGTTGGCTTCTCGCATCTATTGAGGAATCCACGTGCTTGAG
Barcode B-30	CAAGCGTTGGCTTCTCGCATCTCAACCACAATCCACGTGCTTGAG
Barcode B-31	CAAGCGTTGGCTTCTCGCATCTGACTAGTAATCCACGTGCTTGAG
Barcode B-32	CAAGCGTTGGCTTCTCGCATCTCAATGGAAATCCACGTGCTTGAG
Barcode B-33	CAAGCGTTGGCTTCTCGCATCTCACTTCGAATCCACGTGCTTGAG
Barcode B-34	CAAGCGTTGGCTTCTCGCATCTCAGCGTTAATCCACGTGCTTGAG
Barcode B-35	CAAGCGTTGGCTTCTCGCATCTCATACCAAATCCACGTGCTTGAG
Barcode B-36	CAAGCGTTGGCTTCTCGCATCTCCAGTTCAATCCACGTGCTTGAG
Barcode B-37	CAAGCGTTGGCTTCTCGCATCTCCGAAGTAATCCACGTGCTTGAG
Barcode B-38	CAAGCGTTGGCTTCTCGCATCTCCGTGAGAATCCACGTGCTTGAG
Barcode B-39	CAAGCGTTGGCTTCTCGCATCTCCTCCTGAATCCACGTGCTTGAG
Barcode B-40	CAAGCGTTGGCTTCTCGCATCTCGAACTTAATCCACGTGCTTGAG
Barcode B-41	CAAGCGTTGGCTTCTCGCATCTCGACTGGAATCCACGTGCTTGAG

Barcode B-42	CAAGCGTTGGCTTCTCGCATCTCGCATACAATCCACGTGCTTGAG
Barcode B-43	CAAGCGTTGGCTTCTCGCATCTCTCAATGAATCCACGTGCTTGAG
Barcode B-44	CAAGCGTTGGCTTCTCGCATCTCTGAGCCAATCCACGTGCTTGAG
Barcode B-45	CAAGCGTTGGCTTCTCGCATCTCTGGCATAATCCACGTGCTTGAG
Barcode B-46	CAAGCGTTGGCTTCTCGCATCTGAATCTGAATCCACGTGCTTGAG
Barcode B-47	CAAGCGTTGGCTTCTCGCATCTCAAGACTAATCCACGTGCTTGAG
Barcode B-48	CAAGCGTTGGCTTCTCGCATCTGAGCTGAAATCCACGTGCTTGAG
Barcode B-49	CAAGCGTTGGCTTCTCGCATCTGATAGACAATCCACGTGCTTGAG
Barcode B-50	CAAGCGTTGGCTTCTCGCATCTGCCACATAATCCACGTGCTTGAG
Barcode B-51	CAAGCGTTGGCTTCTCGCATCTGCGAGTAAATCCACGTGCTTGAG
Barcode B-52	CAAGCGTTGGCTTCTCGCATCTGCTAACGAATCCACGTGCTTGAG
Barcode B-53	CAAGCGTTGGCTTCTCGCATCTGCTCGGTAATCCACGTGCTTGAG
Barcode B-54	CAAGCGTTGGCTTCTCGCATCTGGAGAACAATCCACGTGCTTGAG
Barcode B-55	CAAGCGTTGGCTTCTCGCATCTGGTGCGAAATCCACGTGCTTGAG
Barcode B-56	CAAGCGTTGGCTTCTCGCATCTGTACGCAAATCCACGTGCTTGAG
Barcode B-57	CAAGCGTTGGCTTCTCGCATCTGTCGTAGAATCCACGTGCTTGAG
Barcode B-58	CAAGCGTTGGCTTCTCGCATCTGTCTGTCAATCCACGTGCTTGAG
Barcode B-59	CAAGCGTTGGCTTCTCGCATCTGTGTTCTAATCCACGTGCTTGAG
Barcode B-60	CAAGCGTTGGCTTCTCGCATCTTAGGATGAATCCACGTGCTTGAG
Barcode B-61	CAAGCGTTGGCTTCTCGCATCTTATCAGCAATCCACGTGCTTGAG
Barcode B-62	CAAGCGTTGGCTTCTCGCATCTTCCGTCTAATCCACGTGCTTGAG
Barcode B-63	CAAGCGTTGGCTTCTCGCATCTTCTTCACAATCCACGTGCTTGAG
Barcode B-64	CAAGCGTTGGCTTCTCGCATCTTGAAGAGAATCCACGTGCTTGAG
Barcode B-65	CAAGCGTTGGCTTCTCGCATCTTGGAACAAATCCACGTGCTTGAG
Barcode B-66	CAAGCGTTGGCTTCTCGCATCTTGGCTTCAATCCACGTGCTTGAG
Barcode B-67	CAAGCGTTGGCTTCTCGCATCTTGGTGGTAATCCACGTGCTTGAG
Barcode B-68	CAAGCGTTGGCTTCTCGCATCTTTCACGCAATCCACGTGCTTGAG
Barcode B-69	CAAGCGTTGGCTTCTCGCATCTAACTCACCATCCACGTGCTTGAG
Barcode B-70	CAAGCGTTGGCTTCTCGCATCTAAGAGATCATCCACGTGCTTGAG
Barcode B-71	CAAGCGTTGGCTTCTCGCATCTAAGGACACATCCACGTGCTTGAG
Barcode B-72	CAAGCGTTGGCTTCTCGCATCTAATCCGTCATCCACGTGCTTGAG
Barcode B-73	CAAGCGTTGGCTTCTCGCATCTAATGTTGCATCCACGTGCTTGAG
Barcode B-74	CAAGCGTTGGCTTCTCGCATCTACACGACCATCCACGTGCTTGAG
Barcode B-75	CAAGCGTTGGCTTCTCGCATCTACAGATTCATCCACGTGCTTGAG
Barcode B-76	CAAGCGTTGGCTTCTCGCATCTAGATGTACATCCACGTGCTTGAG
Barcode B-77	CAAGCGTTGGCTTCTCGCATCTAGCACCTCATCCACGTGCTTGAG
Barcode B-78	CAAGCGTTGGCTTCTCGCATCTAGCCATGCATCCACGTGCTTGAG
Barcode B-79	CAAGCGTTGGCTTCTCGCATCTAGGCTAACATCCACGTGCTTGAG
Barcode B-80	CAAGCGTTGGCTTCTCGCATCTATAGCGACATCCACGTGCTTGAG
Barcode B-81	CAAGCGTTGGCTTCTCGCATCTATCATTCCATCCACGTGCTTGAG
Barcode B-82	CAAGCGTTGGCTTCTCGCATCTATTGGCTCATCCACGTGCTTGAG
Barcode B-83	CAAGCGTTGGCTTCTCGCATCTCAAGGAGCATCCACGTGCTTGAG
Barcode B-84	CAAGCGTTGGCTTCTCGCATCTCACCTTACATCCACGTGCTTGAG

Barcode B-85	CAAGCGTTGGCTTCTCGCATCTCCATCCTCATCCACGTGCTTGAG
Barcode B-86	CAAGCGTTGGCTTCTCGCATCTCCGACAACATCCACGTGCTTGAG
Barcode B-87	CAAGCGTTGGCTTCTCGCATCTCCTAATCCATCCACGTGCTTGAG
Barcode B-88	CAAGCGTTGGCTTCTCGCATCTCCTCTATCATCCACGTGCTTGAG
Barcode B-89	CAAGCGTTGGCTTCTCGCATCTCGACACACATCCACGTGCTTGAG
Barcode B-90	CAAGCGTTGGCTTCTCGCATCTCGGATTGCATCCACGTGCTTGAG
Barcode B-91	CAAGCGTTGGCTTCTCGCATCTCTAAGGTCATCCACGTGCTTGAG
Barcode B-92	CAAGCGTTGGCTTCTCGCATCTGAACAGGCATCCACGTGCTTGAG
Barcode B-93	CAAGCGTTGGCTTCTCGCATCTGACAGTGCATCCACGTGCTTGAG
Barcode B-94	CAAGCGTTGGCTTCTCGCATCTGAGTTAGCATCCACGTGCTTGAG
Barcode B-95	CAAGCGTTGGCTTCTCGCATCTGATGAATCATCCACGTGCTTGAG
Barcode B-96	CAAGCGTTGGCTTCTCGCATCTGCCAAGACATCCACGTGCTTGAG
Barcode B-97	CAAGCGTTGGCTTCTCGCATCTCGGAAGAAATCCACGTGCTTGAG
Barcode B-98	CAAGCGTTGGCTTCTCGCATCTGTGACAAGATCCACGTGCTTGAG
Barcode B-99	CAAGCGTTGGCTTCTCGCATCTGAACCAGAATCCACGTGCTTGAG
Barcode B-100	CAAGCGTTGGCTTCTCGCATCTTTGCTGGAATCCACGTGCTTGAG

Supplementary Table 7. Chemicals and reagents.

Name	Catalog number	Vender
Formaldehyde solution	PI28906	Thermo Fisher Scientific
HEPES pH 7.5	BBH-75-250	Boston BioProducts
Glycine	50046	Sigma-Aldrich
NaCl	AM9760G	Thermo Fisher Scientific
Digitonin	G9441	Promega
$MgCl_2$	AM9530G	Thermo Fisher Scientific
Spermidine	S0266	Sigma-Aldrich
EDTA-free Protease Inhibitor Cocktail	11873580001	Millipore Sigma
NP40	11332473001	Sigma-Aldrich
EDTA Solution pH 8.0	AB00502	AmericanBio
Bovine Serum Albumin (BSA)	A8806	Sigma-Aldrich
Anti-H3K27ac antibody	ab177178	Abcam
Anti-H3K27me3 antibody	9733	Cell Signaling Technology
Histone H3K4me3 antibody	39159	Active Motif
Secondary antibody (Guinea Pig anti-Rabbit IgG)	ABIN101961	Antibodies-Online
pA-Tn5 Transposase – unloaded	C01070002	Diagenode
Triton X-100	T8787	Sigma-Aldrich
T4 DNA Ligase	M0202L	New England Biolabs
T4 DNA Ligase Reaction Buffer	B0202S	New England Biolabs
NEBuffer 3.1	B7203S	New England Biolabs
DPBS	14190144	Thermo Fisher Scientific
Proteinase K	EO0491	Thermo Fisher Scientific
Ampure XP beads	A63880	Beckman Coulter
NEBNext High-Fidelity 2X PCR Master Mix	M0541L	New England Biolabs
SYBR Green I Nucleic Acid Gel Stain	S7563	Thermo Fisher Scientific
DNA Clean & Concentrator-5	D4014	Zymo Research
Tn5 Transposase - unloaded	C01070010	Diagenode
Tagmentation Buffer (2x)	C01019043	Diagenode
Sodium dodecyl sulfate	71736	Sigma-Aldrich
Maxima H Minus Reverse Transcriptase (200 U/L)	EP0751	Thermo Fisher Scientific
dNTP mix	R0192	Thermo Fisher Scientific
SUPERased In RNase Inhibitor	AM2694	Thermo Fisher Scientific
Ampure XP beads	A63880	Beckman Coulter
Dynabeads MyOne C1	65001	Thermo Fisher Scientific
RNase Inhibitor	Y9240L	Enzymatics
Kapa Hotstart HiFi ReadyMix	KK2601	Kapa Biosystems
Nextera XT DNA Preparation Kit	FC-131-1024	Illumina
