# Supplementary material for: Spatial epigenome–transcriptome co-profiling of mammalian tissues
Source: Nature. 2023 Mar 15;616(7955):113–22. doi: 10.1038/s41586-023-05795-1 (PMC10076218; doi:10.1038/s41586-023-05795-1)
Supplement: Supplementary file 2 — Reporting Summary [file 41586_2023_5795_MOESM2_ESM.pdf]

nature research

Corresponding author(s): Rong Fan

Last updated by author(s): Jan 18, 2023

Reporting Summary

Nature Research wishes to improve the reproducibility of the work that we publish. This form provides structure for consistency and transparency in reporting. For further information on Nature Research policies, see our Editorial Policies and the Editorial Policy Checklist.

Statistics

For all statistical analyses, confirm that the following items are present in the figure legend, table legend, main text, or Methods section.

n/a	Confirmed	
☐	☒	The exact sample size (*n*) for each experimental group/condition, given as a discrete number and unit of measurement
☐	☒	A statement on whether measurements were taken from distinct samples or whether the same sample was measured repeatedly
☐	☒	The statistical test(s) used AND whether they are one- or two-sided<br>*Only common tests should be described solely by name; describe more complex techniques in the Methods section.*
☐	☒	A description of all covariates tested
☐	☒	A description of any assumptions or corrections, such as tests of normality and adjustment for multiple comparisons
☐	☒	A full description of the statistical parameters including central tendency (e.g. means) or other basic estimates (e.g. regression coefficient) AND variation (e.g. standard deviation) or associated estimates of uncertainty (e.g. confidence intervals)
☐	☒	For null hypothesis testing, the test statistic (e.g. *F*, *t*, *r*) with confidence intervals, effect sizes, degrees of freedom and *P* value noted<br>*Give P values as exact values whenever suitable.*
☒	☐	For Bayesian analysis, information on the choice of priors and Markov chain Monte Carlo settings
☒	☐	For hierarchical and complex designs, identification of the appropriate level for tests and full reporting of outcomes
☐	☒	Estimates of effect sizes (e.g. Cohen's *d*, Pearson's *r*), indicating how they were calculated

Our web collection on statistics for biologists contains articles on many of the points above.

Software and code

Policy information about availability of computer code

Data collection	EVOS FL Auto 2 Software Revision 2.0.2094.0, Illumina NovaSeq 6000 System.
Data analysis	R 4.1, ArchR 1.0.1, Seurat 4.1, Cell Ranger ATAC 1.2, clusterProfiler 4.2, GREAT 4.0.4, Matlab 2020b, Signac 1.8, Monocle 2.26, ST pipeline 1.7.2<br><br>Scripts for data analysis were written in R and python with code available at https://github.com/di-0579/Spatial_epigenome-transcriptome_co-sequencing and archived at Zenodo (https://doi.org/10.5281/zenodo.7395313), and https://github.com/edicliuyang/Hiplex_proteome

For manuscripts utilizing custom algorithms or software that are central to the research but not yet described in published literature, software must be made available to editors and reviewers. We strongly encourage code deposition in a community repository (e.g. GitHub). See the Nature Research guidelines for submitting code & software for further information.

Data

Policy information about availability of data

All manuscripts must include a data availability statement. This statement should provide the following information, where applicable:

- Accession codes, unique identifiers, or web links for publicly available datasets
- A list of figures that have associated raw data
- A description of any restrictions on data availability

Raw and processed data reported in this paper are deposited in the Gene Expression Omnibus (GEO) with accession code GSE205055. These datasets are available as webresources and can be browsed within the tissue spatial coordinates in UCSC Cell and Genome Browser (https://brain-spatial-omics.cells.ucsc.edu) and our own data portal generated with AtlasXplore (https://web.atlasxomics.com/visualization/Fan).

The resulting fastq files were aligned to the human reference genome (GRCh38) (https://hgdownload.soe.ucsc.edu/goldenPath/hg38/chromosomes/) or mouse reference genome (GRCm38) (https://hgdownload.soe.ucsc.edu/goldenPath/mm10/chromosomes/).

Published data for integration and quality comparison are available online: ENCODE ATAC-seq (E13.5 mouse embryo) (https://www.encodeproject.org/search/?type=Experiment&status=released&related_series.@type=OrganismDevelopmentSeries&replicates.library.biosample.organism.scientific_name=Mus+musculus&assay_title=ATAC-seq&life_stage_age=embryonic%2013.5%20days), ENCODE RNA-seq (E13.5 mouse embryo): Forebrain (https://www.ncbi.nlm.nih.gov/geo/query/acc.cgi?acc=GSE78493);
Midbrain (https://www.ncbi.nlm.nih.gov/geo/query/acc.cgi?acc=GSE78456); Hindbrain (https://www.ncbi.nlm.nih.gov/geo/query/acc.cgi?acc=GSE78481), Mouse organogenesis cell atlas (MOCA) (https://oncoscape.v3.sttrcancer.org/atlas.gs.washington.edu.mouse.rna/downloads), Atlas of gene regulatory elements in adult mouse cerebrum (http://catlas.org/mousebrain/#!/downloads), Atlas of the Adolescent Mouse Brain (http://mousebrain.org/adolescent/downloads.html), Mouse brain scCUT&Tag H3K27ac data (https://www.ncbi.nlm.nih.gov/geo/query/acc.cgi?acc=GSM5949207), Mouse brain scCUT&Tag H3K27me3 data (https://www.ncbi.nlm.nih.gov/geo/query/acc.cgi?acc=GSM5949205), Mouse brain scCUT&Tag H3K4me3 data (https://www.ncbi.nlm.nih.gov/geo/query/acc.cgi?acc=GSE163532), Human hippocampus (snRNA-seq) (https://www.ncbi.nlm.nih.gov/geo/query/acc.cgi?acc=GSE186538), Human hippocampus (scATAC-seq) (https://www.ncbi.nlm.nih.gov/geo/query/a cc.cgi?acc=GSE147672).

Field-specific reporting

Please select the one below that is the best fit for your research. If you are not sure, read the appropriate sections before making your selection.

☒ Life sciences ☐ Behavioural & social sciences ☐ Ecological, evolutionary & environmental sciences

For a reference copy of the document with all sections, see nature.com/documents/nr-reporting-summary-flat.pdf

Life sciences study design

All studies must disclose on these points even when the disclosure is negative.

Sample size	No directly relevant. No sample size calculation was performed. Samples sizes were chosen primarily based on experiment length, sample availability, and sequencing costs. The current manuscript mainly described the new methods for spatial epigenome-transcriptome co-profiling, the sample sizes are sufficient because each sample serves as a proof-of-concept for the new technologies.
Data exclusions	No data were excluded from the study.
Replication	All attempts at replication were successful. For spatial-ATAC-RNA-seq, two biological replicates have been done on P21 mouse brain to verify the reproducibility of the new technology. For spatial-CUT&Tag(H3K27ac)-RNA-seq, two biological replicates have been done on P21 mouse brain to verify the reproducibility the new technology. Other experiments were performed once to serve as a proof-of-concept for the new technologies.
Randomization	Randomization was not applicable because the focus of this paper is the development of new technologies for spatial epigenome-transcriptome co-profiling, and did not involve allocating samples/organisms/participants into experimental groups.
Blinding	Blinding was not applicable because the focus of this paper is the development of new technologies for spatial epigenome-transcriptome co-profiling, and did not involve group allocation, and by extension, blinding.

Reporting for specific materials, systems and methods

We require information from authors about some types of materials, experimental systems and methods used in many studies. Here, indicate whether each material, system or method listed is relevant to your study. If you are not sure if a list item applies to your research, read the appropriate section before selecting a response.

Materials & experimental systems

n/a	Involved in the study	
☐	☒	Antibodies
☒	☐	Eukaryotic cell lines
☒	☐	Palaeontology and archaeology
☐	☒	Animals and other organisms
☐	☒	Human research participants
☒	☐	Clinical data
☒	☐	Dual use research of concern

Methods

n/a	Involved in the study	
☒	☐	ChIP-seq
☒	☐	Flow cytometry
☒	☐	MRI-based neuroimaging

Antibodies

Antibodies used	Anti-H3K27ac antibody (clone number: EP16602), ab177178, Abcam; Secondary antibody (Guinea Pig anti-Rabbit IgG (Heavy & Light Chain) Antibody), ABIN101961, Antibodies-Online; Histone H3K4me3 antibody (pAb), 39159, Active Motif; Anti-H3K27me3 antibody (clone number: C36B11), 9733, Cell Signaling Technology.

Validation

Rabbit monoclonal [EP16602] to Histone H3 (acetyl K27)-ChIP Grade (ab177178), abcam, citation: Science 375, 681–686 (2022); Nat Commun 12:4618 (2021); Front Oncol 11:572585 (2021); Cell Death Dis 12:245 (2021); Cancer Cell 38:334-349.e9 (2020), et al. Please refer to manufacturer's description: https://www.abcam.com/histone-h3-acetyl-k27-antibody-ep16602-chip-grade-ab177178.html?productWallTab=ShowAll
Histone H3K4me3 antibody (pAb), 39159, Active Motif, This antibody has been validated for CUT&Tag using Active Motif's CUT&Tag-IT™ Assay Kit, Catalog No. 53160. Please refer to manufacturer's description: https://www.activemotif.com/catalog/details/39159
Tri-Methyl-Histone H3 (Lys27) (C36B11) Rabbit mAb #9733, Cell Signaling Technology, citation: Nat Cancer 3(9):1071-1087 (2022); Nat Commun 13(1):5883 (2022); Cancer Discov 12(7):1760-1781 (2022), et al. Please refer to manufacturer's description: https://www.cellsignal.com/products/primary-antibodies/tri-methyl-histone-h3-lys27-c36b11-rabbit-mab/9733
Guinea Pig anti-Rabbit IgG (Heavy & Light Chain) Antibody - Preadsorbed, Antibodies-Online, According to the manufacturer's description: ABIN101961 is tested via ELISA to ensure that the titer against the antigen (Rb IgG) is above a certain threshold. We also test to make sure the titer against potentially cross-reactive human IgG, goat IgG, and mouse IgG is below a certain threshold. In addition, we test ABIN101961 against anti-guinea pig Serum, rabbit IgG, and rabbit serum in an immunoelectrophoresis assay. Please refer to manufacturer's description: https://www.antibodies-online.com/antibody/101961/Guinea+Pig+anti-Rabbit+IgG+Heavy++Light+Chain+antibody+-+Preadsorbed/

Animals and other organisms

Policy information about studies involving animals; ARRIVE guidelines recommended for reporting animal research

Laboratory animals

The mouse line Sox10:Cre-RCE:LoxP (EGFP), on a C57BL/6xCD1 mixed genetic background. Animals of both sexes were used for experiments at P21/P22.
P22 (spatial-ATAC-RNA-Seq, 100 barcodes), Male
P22 (spatial-CUT&Tag(H3K27ac)-RNA-Seq, 100 barcodes), Female
P22 (spatial-CUT&Tag(H3K27me3)-RNA-Seq, 100 barcodes), Male
P22 (spatial-CUT&Tag(H3K4me3)-RNA-Seq, 100 barcodes), Female
P21 (spatial-ATAC-RNA-Seq, 50 barcodes, replica 1), Male
P21 (spatial-CUT&Tag(H3K27ac)-RNA-Seq, 50 barcodes, replica 1), Male
P21 (spatial-ATAC-RNA-Seq, 50 barcodes, replica 2), Female
P21 (spatial-CUT&Tag(H3K27ac)-RNA-Seq, 50 barcodes, replica 2), Female

All animals were free from mouse bacterial and viral pathogens, ectoparasites and endoparasites. The following light/dark cycle was kept for the mice: dawn 6:00-7:00, daylight 7:00-18:00, dusk 18:00-19:00, night 19:00-6:00. Mice were housed in individually ventilated cages with a maximum number of 5 per cage (IVC sealsafe GM500, tecniplast). General housing parameters such as temperature, ventilation, and relative humidity followed the European convention for the protection of vertebrate animals used for experimental and other scientific purposes. The air quality was controlled by using the stand-alone air handling units equipped with a HEPA filter. The consistent relative air humidity was 55%±10 with a temperature of 22 °C. The husbandry parameters were monitored with ScanClime® (Scanbur) units. The cages contained card box shelter, gnawing sticks, and nesting material (Scanbur), placed on a hardwood bedding (TAPVEI, Estonia). The mice were provided a regular chow diet and water was supplied with a water bottle and changed weekly. Cages were changed every two weeks in a laminar air-flow cabinet.

Wild animals

No wild animals were used in the study.

Field-collected samples

No field collected samples were used in the study.

Ethics oversight

All experimental procedures were conducted following the European directive 2010/63/EU, local Swedish directive L150/SJVFS/2019:9, Saknr L150, and Karolinska Institutet complementary guidelines for procurement and use of laboratory animals, Dnr. 1937/03-640. All the procedures described were approved by the local committee for ethical experiments on laboratory animals in Sweden (Stockholms Norra Djurförsöksetiska nämnd), lic. nr. 1995/2019 and 7029/2020.

Note that full information on the approval of the study protocol must also be provided in the manuscript.

Human research participants

Policy information about studies involving human research participants

Population characteristics

31-year-old Caucasian male, with no psychiatric or neurological diagnosis, toxicology negative for psychotropic medications and drugs.

Recruitment

We analyzed brain hippocampus tissue from a 31 year-old Caucasian male, with postmortem interval (PMI, time from demise to brain collection) of 6.5 hours, with no psychiatric or neurological diagnosis, who died of a traumatic accident, and had a high global functioning before death as measured by Global Assessment Scale (GAS) score which was 90 (score 1 to 100, with 100 the highest functioning), and with toxicology negative for psychotropic medications and drugs. Biases were not applicable because the focus of this paper is the development of the new methods for spatial epigenome-transcriptome co-profiling, and each sample serves as a proof-of-concept for the new technologies.

Ethics oversight

The human brain tissue was obtained from the Brain Collection of the New York State Psychiatric Institute (NYSPI) at Columbia University, which includes brain samples from the Republic of Macedonia. Brain tissue collection was conducted with NYSPI Institutional Review Board approval and informed consent obtained from next of kin who agreed to donate the brains and participated in psychological autopsy interviews.

Note that full information on the approval of the study protocol must also be provided in the manuscript.
